# Inhibition of DPAGT1 suppresses HER2 shedding and trastuzumab resistance in human breast cancer

**DOI:** 10.1172/JCI164428

**Published:** 2023-07-17

**Authors:** Muwen Yang, Li, Lingzhi Kong, Shumei Huang, Lixin He, Pian Liu, Shuang Mo, Xiuqing Lu, Xi Lin, Yunyun Xiao, Dongni Shi, Xinjian Huang, Boyu Chen, Xiangfu Chen, Ying Ouyang, Li, Chuyong Lin, Libing Song

**Affiliations:** 1 Department of Experimental Research, State Key Laboratory of Oncology in South China, Collaborative Innovation Center for Cancer Medicine and; 2 Department of Biochemistry, Zhongshan School of Medicine, Sun Yat-sen University, Guangzhou, Guangdong China.; 3 Cancer Center, Union Hospital, Tongji Medical College, Huazhong University of Science and Technology, Wuhan, China.; 4 Key Laboratory of Protein Modification and Degradation, School of Basic Medical Sciences, Guangzhou Institute of Oncology, Tumor Hospital, Guangzhou Medical University, Guangzhou, China.

**Keywords:** Oncology, Breast cancer, Drug therapy, Molecular biology

## Abstract

Human epidermal growth factor receptor 2–targeted (HER2-targeted) therapy is the mainstay of treatment for HER2^+^ breast cancer. However, the proteolytic cleavage of HER2, or HER2 shedding, induces the release of the target epitope at the ectodomain (ECD) and the generation of a constitutively active intracellular fragment (p95HER2), impeding the effectiveness of anti-HER2 therapy. Therefore, identifying key regulators in HER2 shedding might provide promising targetable vulnerabilities against resistance. In the current study, we found that upregulation of dolichyl-phosphate N-acetylglucosaminyltransferase (DPAGT1) sustained high-level HER2 shedding to confer trastuzumab resistance, which was associated with poor clinical outcomes. Upon trastuzumab treatment, the membrane-bound DPAGT1 protein was endocytosed via the caveolae pathway and retrogradely transported to the ER, where DPAGT1 induced N-glycosylation of the sheddase — ADAM metallopeptidase domain 10 (ADAM10) — to ensure its expression, maturation, and activation. N-glycosylation of ADAM10 at N267 protected itself from ER-associated protein degradation and was essential for DPAGT1-mediated HER2 shedding and trastuzumab resistance. Importantly, inhibition of DPAGT1 with tunicamycin acted synergistically with trastuzumab treatment to block HER2 signaling and reverse resistance. These findings reveal a prominent mechanism for HER2 shedding and suggest that targeting DPAGT1 might be a promising strategy against trastuzumab-resistant breast cancer.

## Graphical abstract

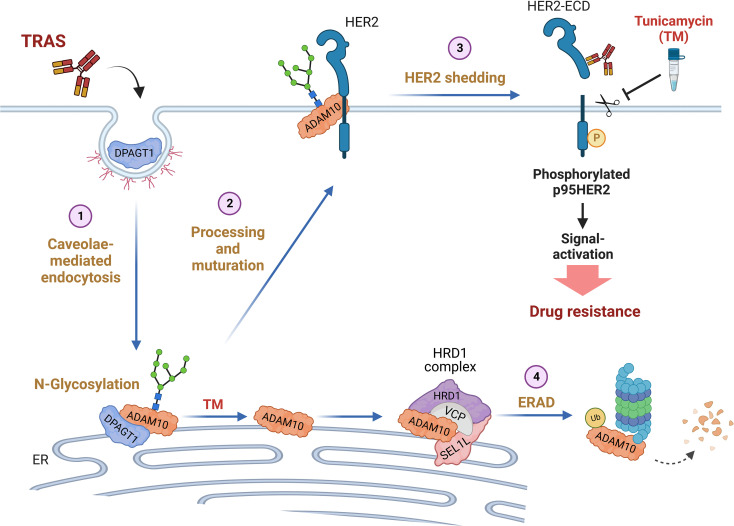

## Introduction

Human epidermal growth factor receptor 2–positive (HER2^+^) breast cancer — referring to breast cancer tumors that have HER2, also known as ERBB2, overexpression and/or amplification — accounts for 20%–30% of all breast cancer cases (1). Notably, HER2^+^ breast cancer is characterized by the sustained activation of HER2 signaling, which has been successfully exploited for selective targeted therapy by monoclonal antibodies, such as trastuzumab (2). Clinically, trastuzumab therapy yields significant survival benefits in patients with HER2^+^ breast cancer (3, 4). However, approximately 15% of patients with early stage HER2^+^ breast cancer experience recurrence after adjuvant trastuzumab therapy (4), and patients with advanced-stage HER2^+^ breast cancer who receive trastuzumab-based palliative therapy will inevitably experience disease progression within approximately 12 months (5). Further, the objective response rate of trastuzumab monotherapy only ranged from 12%–34% (6–8).

Ectodomain (ECD) shedding, which induces the proteolytic cleavage of the extracellular domain of proteins (9, 10), plays an important role in regulating the activity of multiple cell surface receptors, including cytokine receptors — tumor necrosis factor receptor 1 (TNFR1) and interleukin 6 receptor (IL6R) — notch receptors, transforming growth factor β (TGFβ) receptor, and receptor tyrosine kinases (RTK) — HER2, HER4, vascular endothelial growth factor receptor 2 (VEGFR2), and MET protooncogene, receptor tyrosine kinase (c-MET) (9). Recently, dysregulation of ECD shedding of transmembrane receptors has been reported to contribute to tumor progression and drug resistance (9). In particular, shedding of the HER2 receptor results in the release of soluble HER2-ECD, which contains the trastuzumab-recognition epitope, but retains the membrane-associated oncogenic p95HER2 fragment (11, 12). Importantly, the p95HER2 fragment in tumor cells exerts higher tyrosine kinase activity to promote cancer cell growth and survival, which greatly contributes to trastuzumab resistance (13, 14). The HER2 shedding has been found in several HER2-overexpressing cancers, including breast cancer, gastric cancer, and uterine serous carcinoma (15–17). Up to 30% of patients with HER2^+^ breast cancer had elevated tumoral p95HER2 or serum HER2-ECD, which were associated with attenuated response to trastuzumab and poor outcomes (14, 18–20). However, the mechanisms for HER2 shedding remain largely enigmatic. Therefore, identifying key regulators involved in HER2 shedding might open new avenues to prevent or overcome trastuzumab resistance.

Dolichyl-phosphate N-acetylglucosaminephosphotransferase 1 (*DPAGT1*), encodes an endoplasmic reticulum (ER) integral membrane enzyme, belonging to the polyprenyl-phosphate N-acetyl hexosamine 1-phosphate transferase (PNPT) family (21). DPAGT1 is involved in catalyzing the first and critical step of precursor oligosaccharide synthesis in the dolichol-linked oligosaccharide pathway during N-linked glycosylation (N-glycosylation), which regulates the properties and bioactivities of many eukaryotic proteins (22). Although it has been reported that DPAGT1 upregulation was associated with tumor aggressiveness in oral cancer and esophageal squamous cell carcinoma (23–25), it remains unclear whether and how DPAGT1 upregulation is involved in breast cancer progression.

In the present study, we found that DPAGT1-mediated N-glycosylation of ADAM10 sheddase at N267 was required for the stability, maturation, and activation of ADAM10. This occurred through the protection of ADAM10 from ER-associated degradation (ERAD), which led to robust HER2 shedding, trastuzumab resistance, and poor clinical outcomes**.** Importantly, inhibition of DPAGT1 resensitized trastuzumab-resistant HER2^+^ breast tumors. Therefore, these findings identified DPAGT1 as a key regulator of HER2 shedding and suggested that DPAGT1 inhibition might be a promising strategy against trastuzumab-resistant HER2^+^ breast cancer.

## Results

### DPAGT1 is a potential regulator of HER2 shedding and correlates with poor prognosis.

**Figure 1:**
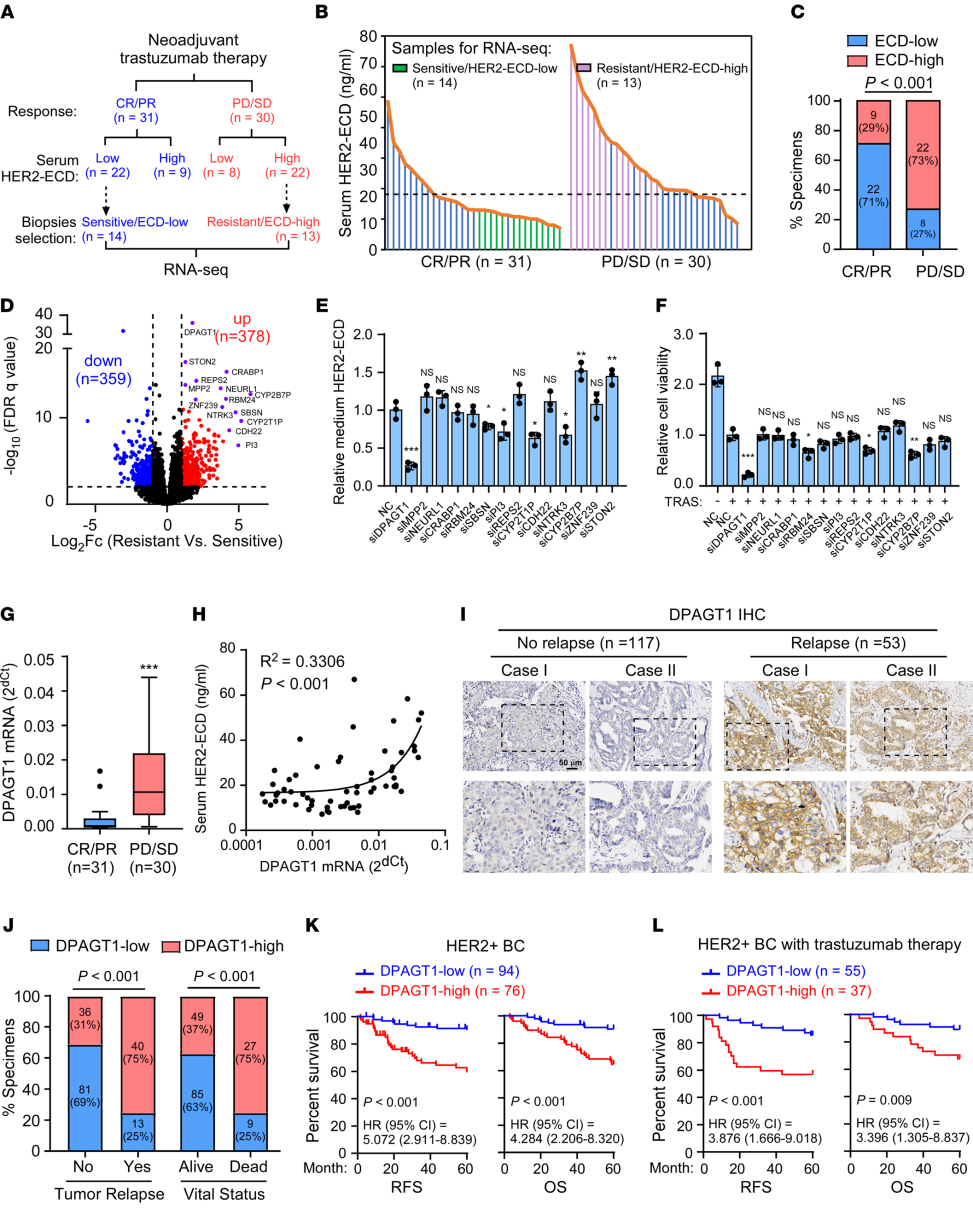
DPAGT1 is a potential regulator of HER2 shedding and correlates with poor prognosis. (**A**) A schematic diagram showing the information regarding neoadjuvant trastuzumab therapy response and serum HER2-ECD expression in 61 patients. Tumor biopsies with trastuzumab-sensitive/ECD-low (*n* = 14) or trastuzumab-resistant/ECD-high (*n* = 13) were selected for RNA-Seq analysis. (**B**) ELISA analysis of HER2-ECD levels in the serum derived from HER2^+^ breast cancer patients before trastuzumab therapy. The data were log_2_ transformed and the median was used as the cutoff. The green and pink histograms indicated the samples selected for RNA-Seq. (**C**) Correlation analysis between serum HER2-ECD level and trastuzumab response. (**D**) Volcano plot showing the gene expression in RNA-Seq analysis in trastuzumab-resistant HER2^+^ breast cancer tissues compared with sensitive ones. FDR < 0.01 and fold change > 2 were used as the cutoff. (**E**) Histograms showing relative HER2-ECD level in the medium of NC- or the indicated siRNA-transfected SK-BR-3 cells. (**F**) Relative cell viability of NC- or the indicated siRNA-transfected SK-BR-3 cells upon trastuzumab treatment (20 μg/mL, 48 hours). (**G**) qRT-PCR analysis of *DPAGT1* mRNA in the 61 HER2^+^ breast cancer patient biopsies. *GAPDH* was used as an internal control. (**H**) Linear regression analysis of the correlation between *DPAGT1* mRNA expression in biopsies and HER2-ECD levels in patient serum. (**I**) Representative IHC staining images of DPAGT1 in HER2^+^ breast cancer specimens. Scale bar: 50 μm. (**J**) Correlation analysis between DPAGT1 expression, tumor relapse, and patient vital status. (**K**) Kaplan-Meier analysis of RFS (left) and OS (right) curves in HER2^+^breast cancer with low- or high-DPAGT1 expression (*n* = 170). HR, hazard ratio. (**L**) Kaplan-Meier curves of RFS (left) and OS (right) in the patient subgroup receiving trastuzumab therapy. Data in **E** and **F** are plotted as the mean ± SD of biological triplicates. Data in **G** are shown in a Tukey Boxplot. A χ^2^ test was used in **C** and **J**. An unpaired 2-sided Student’s *t* test was used in **E**–**G**. A Log-rank test was used in **K**–**L**. *P < 0.05, **P < 0.01, ***P < 0.001.

To identify potential regulators of HER2 shedding in trastuzumab resistance, peripheral blood samples and breast cancer biopsies were collected from 61 patients with locally advanced or early stage HER2^+^ breast cancer (with “early stage” defined as lymph node–positive or tumor size over 5 cm in diameter) before they received neoadjuvant trastuzumab therapy. The serum HER2-ECD level was determined to stratify the subgroups from patients with sensitive or poor responses using the median as the cutoff value for high or low serum HER2-ECD expression (Figure 1, A and B). Statistical analysis revealed that patients with high-serum HER2-ECD were prone to be refractory to trastuzumab (*P* < 0.001), and that, among the patients with complete response (CR) or partial response (PR) after trastuzumab therapy, 71% (22 of 31) had low serum HER2-ECD, while 73% (22 of 30) of the patients with progressive disease (PD) or stable disease (SD) expressed high serum HER2-ECD levels (Figure 1, A–C and Supplemental Figure 1A; supplemental material available online with this article; https://doi.org/10.1172/JCI164428DS1). These results indicated that high serum HER2-ECD level was associated with poor trastuzumab response and worse progression in HER2^+^ breast cancer.

To identify potential regulators of HER2 shedding, the biopsies from the half of the resistant (PD/SD) group with the highest levels of HER2-ECD levels and the half of the sensitive (CR/PR) group with the lowest HER2-ECD levels were selected for RNA-Seq. Among these biopsies, RNA samples of 27 biopsies that met the quality and dose demand for RNA sequencing were finally used. The RNA-Seq analysis indicated that 378 genes were upregulated while 359 genes were downregulated in the resistant group compared with that in the sensitive group (Figure 1D). Furthermore, the most significantly upregulated 14 genes were tested for their effect on HER2 shedding and trastuzumab resistance (Supplemental Figure 1B). As shown in Figure 1, E and F and Supplemental Figure 1C, silencing of *DPAGT1* resulted in the most significant reduction of HER2-ECD level in the culture medium and inhibition of trastuzumab-induced cell death (Figure 1, E and F and Supplemental Figure 1C), which suggested that DPAGT1 might contribute to HER2 shedding and trastuzumab resistance. In line with this hypothesis, quantitative real-time reverse transcription PCR (qRT-PCR) analysis of the 61 HER2^+^ breast cancer biopsies showed that *DPAGT1* mRNA expression was significantly upregulated in trastuzumab-resistant tissues compared with sensitive tissues and was significantly associated with the serum HER2-ECD level (R^2^ = 0.3306, *P* < 0.001) in these patients (Figure 1, G and H). Interestingly, *DPAGT1* mRNA expression correlated positively with the serum HER2-ECD in the resistant group (*P* = 0.012) but not in the sensitive group (*P* = 0.073) (Supplemental Figure 1D). These results provided a potential clinical link between DPAGT1 expression and HER2 shedding in the HER2^+^ breast cancer.

The clinical significance of DPAGT1 protein levels was further examined in 170 paraffin-embedded (FFPE) HER2^+^ breast cancer specimens (Supplemental Table 1). IHC staining analysis revealed that the expression of DPAGT1 was markedly increased in the pretherapeutic HER2^+^ breast tumors from patients who had recurrence within 5 years after surgery compared with that of patients without tumor recurrence (Figure 1I). Significantly, high DPAGT1 expression was strongly correlated with tumor relapse, patient death, and several clinicopathological characteristics, including advanced stages and higher Ki67 expression (Figure 1J and Supplemental Table 2). Importantly, high DPAGT1 expression was inversely associated with relapse-free survival (RFS) and overall survival (OS) in patients with HER2^+^ breast cancer and in patients with HER2^+^ breast cancer who received trastuzumab treatment (Figure 1, K and L). It was also recognized as one of the independent prognostic factors in patients with HER2^+^ breast cancer (Supplemental Figure 1E). In addition, The Cancer Genome Atlas (TCGA) data analysis showed a universal increase of *DPAGT1* expression in diverse HER2-overexpressing human cancers (26) (Supplemental Figure 1F). Taken together, these results indicated that DPAGT1 upregulation correlated with poor prognosis in patients with HER2^+^ breast cancer.

### DPAGT1 induces HER2 shedding and trastuzumab resistance.

**Figure 2:**
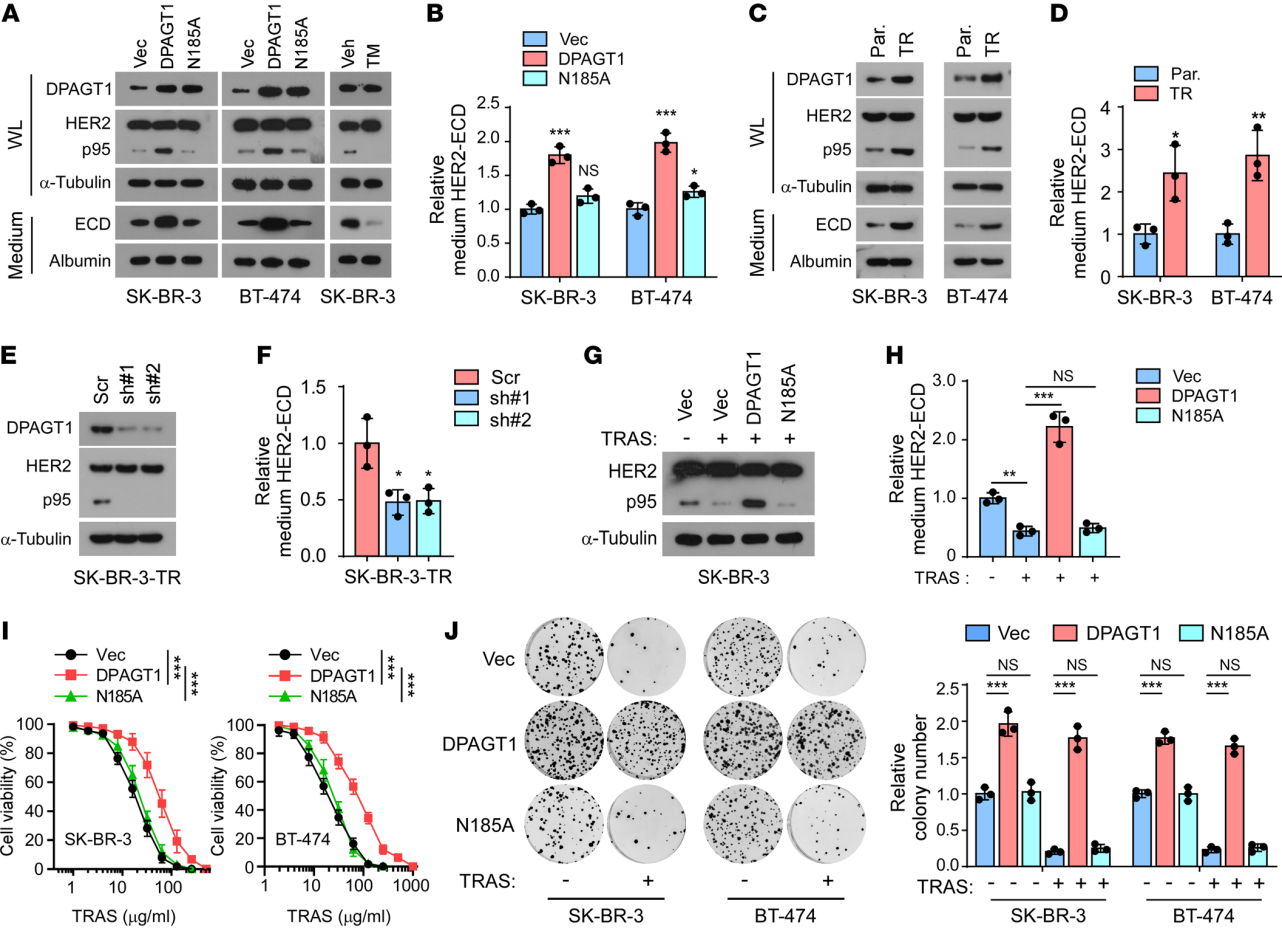
DPAGT1 induces HER2 shedding and trastuzumab resistance. (**A**) IB analysis showing the expression of DPAGT1, HER2, and p95HER2 in the whole lysate (WL) and HER2-ECD in the medium. α-Tubulin was used as a loading control for the WL and Albumin was used as a loading control for proteins in the medium. (**B**) ELISA analysis of HER2-ECD levels in the medium from indicated cells. (**C**) IB analysis of expression of HER2 and p95HER2 in the whole lysate (WL) and the ECD level in condensed culture medium from the parental (Par.) and trastuzumab-resistant (TR) SK-BR-3 and BT-474 cells. (**D**) Relative HER2-ECD level in the medium from the parental and trastuzumab-resistant cells. (**E**) IB analysis of HER2 and p95HER2 expression in the indicated trastuzumab-resistant SK-BR-3 cells. α-Tubulin was used as a loading control. (**F**) Relative HER2-ECD level in the medium from control or DPAGT1-silenced trastuzumab-resistant SK-BR-3 cells. (**G**) IB analysis of HER2 and p95HER2 expression in the Vector-, DPAGT1-, or DPAGT1-N185A-transduced SK-BR-3 cells with or without trastuzumab treatment (20 μg/mL). α-Tubulin was used as a loading control. (**H**) Relative HER2-ECD level in the medium from indicated SK-BR-3 cells. (**I**) Cell viability assay analyzing the sensitivity of the indicated SK-BR-3 and BT-474 cells to trastuzumab treatment (20 μg/mL, 48 hours). (**J**) Representative image (left) and quantification (right) of surviving colonies formed by the indicated SK-BR-3 and BT-474 cells with or without trastuzumab treatment (20 μg/mL). Data in **B**, **D**, **F**, **H**, **I**, and **J** were plotted as the mean ± SD of biological triplicates. An unpaired 2-sided Student’s *t* test was used in **B**, **D**, **F**, **H**, and **J**. 2-way ANOVA was used in **I**. *P < 0.05, **P < 0.01, ***P < 0.001.

The biological role of DPAGT1 in the proteolytic cleavage of HER2 was further investigated. As shown in Figure 2, A and B, overexpression of DPAGT1, but not the enzyme-dead mutant DPAGT1-N185A (21), dramatically increased the level of p95HER2 in HER2^+^ breast cancer cells and the HER2-ECD level in the conditional medium, which was abolished by DPAGT1 inhibition with tunicamycin (TM). These results suggested that the N-glycosylation activity of DPAGT1 was indispensable for HER2 shedding. Consistently, the trastuzumab-resistant SK-BR-3 and BT-474 cell lines exhibited elevated expression levels of DPAGT1, p95HER2, and HER2-ECD compared with those in the parental cells (Figure 2, C and D). Furthermore, knockdown of *DPAGT1* reduced the levels of p95HER2 and HER2-ECD in the trastuzumab-resistant SK-BR-3 cells (Figure 2, E and F). Moreover, we found that the repressive effect of trastuzumab on HER2 shedding, which was consistent with a previous report (27), was dramatically abrogated by ectopic expression of *DPAGT1*, as indicated by sustained high levels of intracellular p95HER2 and HER2-ECD in the conditional medium from trastuzumab-treated *DPAGT1*-overexpressing cells, but not in the cells overexpressing DPAGT1-N185A mutant (Figure 2, G and H). Therefore, these results indicated that upregulation of DPAGT1 promoted HER2 shedding in HER2^+^ breast cancer cells.

In agreement with the promotive effect of DPAGT1 on HER2 shedding, overexpressing of *DPAGT1* drastically impaired the sensitivity of HER2^+^ breast cancer cells to trastuzumab treatment, as indicated by increased cell viability and colony formation of HER2^+^ breast cancer cells, as well as the elevated activity of HER2 downstream effector protein kinase B (AKT) and extracellular regulated kinase (ERK1/2) (Figure 2, I and J and Supplemental Figure 2, A and B). However, we did not observe the resistant effect on trastuzumab treatment in the DPAGT1-N185A mutant-overexpressing cells (Figure 2, I and J and Supplemental Figure 2, A and B). A similar resistant effect was also observed in *DPAGT1*-overexpressed cells treated with pertuzumab, which also binds to the extracellular moiety of HER2 (Supplemental Figure 2C). Therefore, these results demonstrated that DPAGT1 induced HER2 shedding and trastuzumab resistance.

### DPAGT1 promotes trastuzumab resistance by inducing HER2 shedding in vivo.

**Figure 3:**
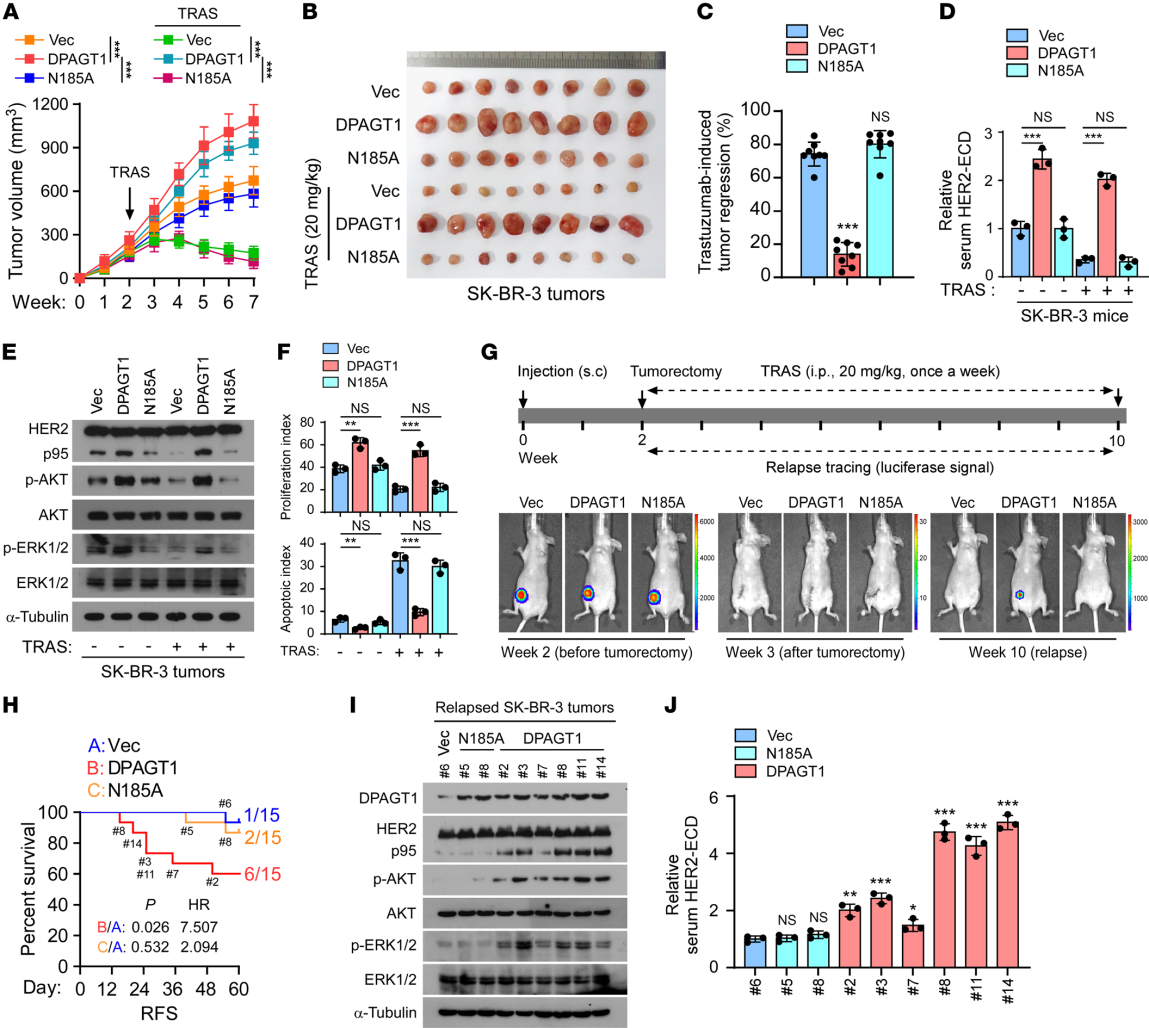
DPAGT1 promotes trastuzumab resistance by inducing HER2 shedding in vivo. (**A**) Tumor growth curves of the xenograft tumors (*n* = 8/group) formed by Vector-, DPAGT1-, or DPAGT1/N185A-transduced SK-BR-3 cells. After 2 weeks of inoculation of the indicated cells, trastuzumab (20 mg/kg) was administrated once a week for 5 weeks. Tumor volumes were assayed weekly. (**B**) Representative pictures of xenograft tumors formed by the indicated cells treated with or without trastuzumab (20 mg/kg). (**C**) The tumor growth inhibition rate induced by trastuzumab in each group was calculated by the reduction in tumor volume after trastuzumab treatment relative to the tumor volume treated with IgG, using the formula (V^IgG^ – V^TRAS+^) / V^IgG^. (**D**) ELISA analysis of HER2-ECD level in serum from the indicated mice. (**E**) IB analysis of the expression of p95HER2, p-AKT, AKT, p-ERK1/2, and ERK1/2 in the tumors formed by the indicated SK-BR-3 cells. (**F**) The proliferation index and apoptotic index, represented as the percentage of Ki67^+^ cells and TUNEL^+^ cells, in the tumors formed by the indicated SK-BR-3 cells. (**G**) A scheme showing the indicated s.c. tumor recurrence model with trastuzumab treatment. (**H**) Kaplan–Meier relapse-free survival of mice (*n* = 15/group) from the Figure 3G indicating the number of mice in each group recurring at the indicated time. HR, hazard ratio. (**I**) IB analysis of expression of p95HER2, p-AKT, AKT, p-ERK1/2, and ERK1/2 in the indicated recurrent tumors from each group. (**J**) ELISA analysis of the serum HER2-ECD level in the mice in each group. Data in **A**, **C**, **D**, **F**, and **J** were plotted as the mean ± SD of biological triplicates. Data in **A** and **C** was plotted as the means ± SD of 8 mice. Data in **D** was plotted as the mean ± SD of 3 mice. An unpaired 2-sided Student’s *t* test was used in **C**, **D**, and **F**. 2-way ANOVA was used in **A**. The log-rank test was used in **H**. *P < 0.05, **P < 0.01, ***P < 0.001.

The role of DPAGT1 in trastuzumab resistance was further investigated in vivo. Briefly, BALB/C-nu mice were s.c. injected with Vector-, DPAGT1-, or DPAGT1-N185A-transduced SK-BR-3 cells. After 2 weeks, the mice in each group were equally subdivided into 2 halves (*n* = 8/subgroup). The mice in each subgroup were given an i.p. injection of either IgG or trastuzumab once a week. Notably, the SK-BR-3/DPAGT1 tumors grew much faster than those of the other 2 groups, regardless of trastuzumab treatment, indicating that DPAGT1 promoted tumor growth and induced trastuzumab resistance in vivo (Figure 3, A and B and Supplemental Figure 3A). To exclude the influence of DPAGT1 expression on tumor growth, the tumor growth inhibition rates induced by trastuzumab were compared. As shown in Figure 3C, the tumor growth inhibition rate induced by trastuzumab in the Vector- or DPAGT1-N185A-transduced SK-BR-3 tumors was above 70% but was drastically decreased to 14% in the SK-BR-3/DPAGT1 tumors. Validation analysis showed that DPAGT1 promoted HER2 proteolytic cleavage and activation of HER2-downstream signaling in tumors, resulting in an increased proliferation index and reduced apoptotic index (Figure 3, D–F and Supplemental Figure 3B).

Moreover, to mimic the trastuzumab therapy in the adjuvant setting, subcutaneous SK-BR-3 tumors were removed when they grew to a volume of approximately 200 mm^3^ and trastuzumab treatment was subsequently administered (Figure 3G). The SK-BR-3 tumor cells were constructed to stably express luciferase; therefore, tumor relapse was traced using the luciferase signal (Figure 3G). No luciferase signal was observed 1 week after the tumorectomy, which indicated successful tumor resection (Figure 3G). Notably, as shown in Figure 3H, 6 of 15 (40%) SK-BR-3/DPAGT1 tumor-removed mice suffered tumor relapse, while the tumor recurrence incidence occurred in only 1 of 15 (6.67%) in the Vector tumor-removed group and 2 of 15 (13.33%) in the DPAGT1-N185A tumor-removed group, respectively. Immunoblotting (IB) analysis showed that the expression levels of p95HER2, phosphorylated-Akt (p-AKT), and p-ERK1/2 in the recurrent DPAGT1-overexpressing tumors, as well as the serum level of HER2-ECD detected by an ELISA, especially in those that recurred earlier, were much higher than those in the recurrent vector mice and recurrent DPAGT1-N185A mice, indicating that the HER2 shedding was associated with the relapse of *DPAGT1*-overexpressing tumors (Figure 3, I and J). Taken together, these results indicate that DPAGT1 rendered trastuzumab resistance, at least partially, by inducing HER2 shedding.

### Trastuzumab induces retrograde transport of DPAGT1.

**Figure 4:**
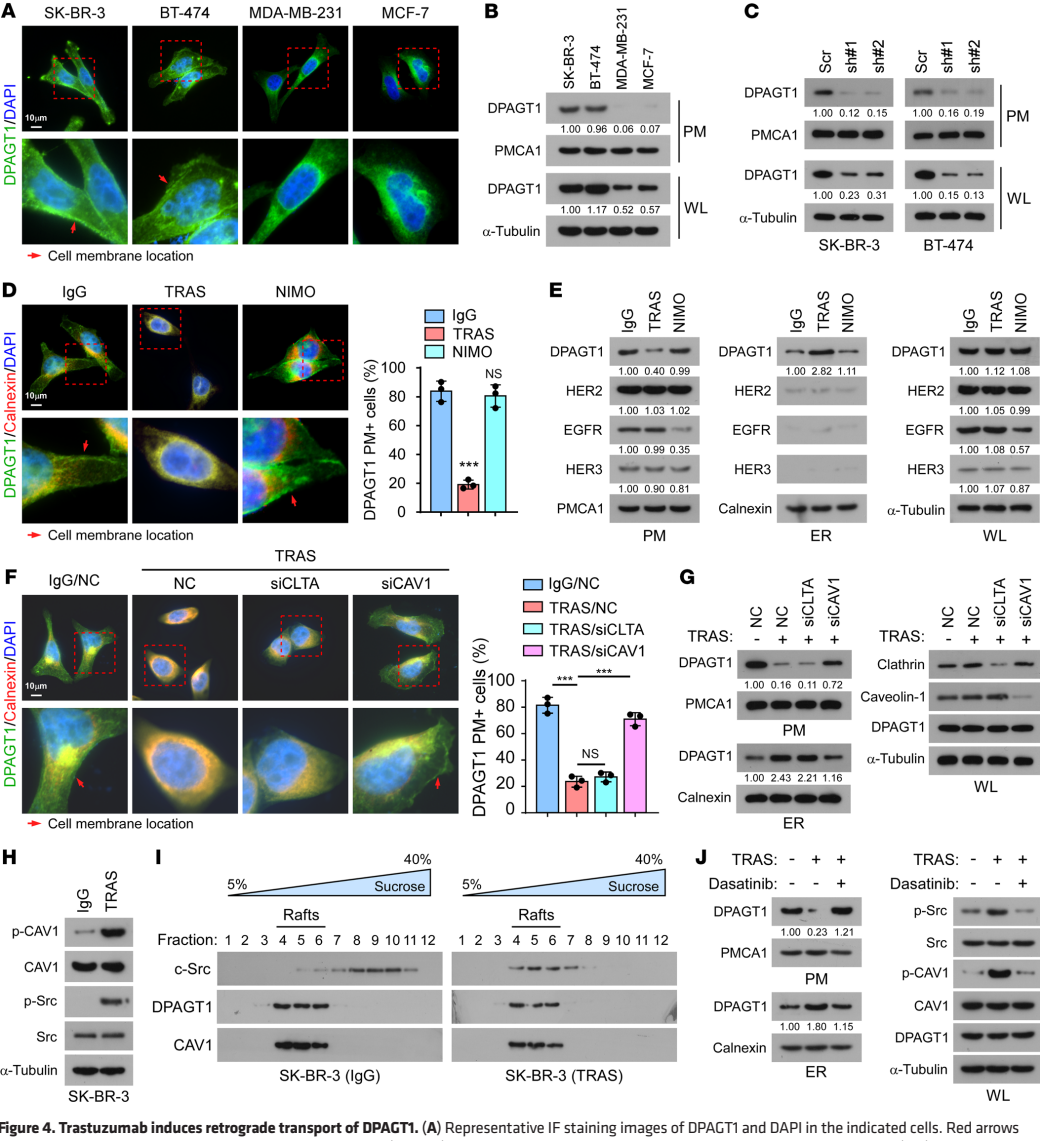
Trastuzumab induces retrograde transport of DPAGT1. (**A**) Representative IF staining images of DPAGT1 and DAPI in the indicated cells. Red arrows indicate the location of the cell membrane. Scale bar: 10 μm (**B** and **C**) IB analysis of DPAGT1 expression in the extracted plasma membrane (PM) and whole lysate (WL) from SK-BR-3, BT-474, MDA-MB-231, and MCF-7 cells (**B**), or from Vector- or DPAGT1-silenced SK-BR-3 and BT-474 cells (**C**). PMCA1 was used as a PM marker. α-Tubulin was used as a loading control. (**D**) Representative IF staining images of DPAGT1 in SK-BR-3 cells treated with IgG, trastuzumab, or nimotuzumab (NIMO, 20 μg/mL). The percentage of DPAGT1 PM^+^ cells was quantified in 10 random fields. Scale bar: 10 μm (**E**) IB analysis of expression of DPAGT1, HER2, EGFR, and HER3 in the extracted PM, extracted ER, and WL of SK-BR-3 cells treated with IgG, trastuzumab, or nimotuzumab. Calnexin was used as an ER marker. PMCA1 was used as a PM marker. α-Tubulin was used as a loading control. (**F**) Representative IF staining image (left) and quantification (right) of DPAGT1 PM^+^ cells in the indicated cells. Scale bar: 10 μm (**G**) IB analysis of DPAGT1 expression in the extracted PM, extracted ER, and WL of indicated SK-BR-3 cells. Calnexin was used as an ER marker. PMCA1 was used as a PM marker. α-Tubulin was used as a loading control. (**H**) IB analysis of expression p-CAV1^Y14^, CAV1, p-c-Src^Y416^, and c-Src in IgG- or trastuzumab-treated SK-BR-3 cells. α-Tubulin was used as a loading control. (**I**) IB analysis of expression of DPAGT1, c-Src, and CAV1 in the lipid rafts isolated from IgG- or trastuzumab-treated SK-BR-3 cells. (**J**) IB analysis of DPAGT1 expression in the extracted PM and ER fractions from SK-BR-3 cells treated with IgG, trastuzumab, or trastuzumab + c-Src inhibitor Dasatinib (4 μM). Data in **D** and **F** were plotted as the mean ± SD of biological triplicates and analyzed with an unpaired 2-sided Student’s *t* test. The arrows in panels **D** and **F** indicate plasma membrane expression. Relative protein expression was quantified by Image J. *P < 0.05, **P < 0.01, ***P < 0.001.

DPAGT1 is an ER-localized protein (21). However, our IHC staining showed that DPAGT1 was also localized at the plasma membrane (PM) in HER2^+^ breast cancer tissues (Figure 1I). Interestingly, immunofluorescence (IF) staining and PM extraction assays showed that HER2^+^ breast cancer cell lines SK-BR-3 and BT-474 displayed clear PM localization of DPAGT1; however, it was rarely detected at the PM of cell lines that are not HER2^+^, such as MDA-MB-231 or MCF-7 (Figure 4, A and B). The observed DPAGT1 protein signal in the PM fraction was DPAGT1-specific instead of an unspecific signal, as it was robustly decreased in DPAGT1-silenced HER2^+^ cells (Figure 4C). We further questioned whether the subcellular location of DPAGT1 could be affected by trastuzumab treatment. As shown in Figure 4, D and E, trastuzumab treatment drastically induced the retrograde transport of DPAGT1, as indicated by an evident decrease in the PM and increase in the ER. However, treatment with an anti-EGFR monoclonal antibody, nimotuzumab, which only recognizes the EGFR extracellular domain, had no effect on DPAGT1 subcellular location, suggesting that the PM/ER translocation of DPAGT1 might be specific to trastuzumab treatment.

We then investigated the mechanism underlying trastuzumab treatment–induced PM/ER translocation of DPAGT1. We first examined whether trastuzumab treatment could induce the PM/ER translocation of HER2 and other HER receptors since HER2 forms homodimers or heterodimers with other HER receptor family members (28). Both IB and flow cytometry analyses showed that the PM, ER, and total expression levels of HER2, EGFR, and HER3, were not affected by trastuzumab treatment in the SK-BR-3 cells (Figure 4E and Supplemental Figure 4A). Although treatment with the EGFR therapeutic monoclonal antibody nimotuzumab substantially decreased the PM and total expression of EGFR, it had no effect on HER2 or HER3 (Figure 4E and Supplemental Figure 4A). These findings indicated that trastuzumab treatment did not induce the PM/ER translocation of HER2, EGFR or HER3. Meanwhile, we found that neither nimotuzumab treatment nor silencing *EGFR* or *HER3* had impact on the PM and ER location of DPAGT1, suggesting that DPAGT1 retrograde transport was not regulated by these 2 HER2 partners (Figure 4E and Supplemental Figure 4B). Furthermore, upon trastuzumab treatment, the more DPAGT1 was expressed in HER2^+^ breast cancer cells, the more DPAGT1 protein would be retrogradely transported from the plasma membrane to the ER (Supplemental Figure 4C).

Notably, a number of studies have provided compelling evidence that, unlike other HER receptor family members, HER2 is endocytosis-resistant, and is thus steadily maintained at the PM even upon antibody stimulation (29–31). It is proposed that the resistance to internalization of HER2 might be caused by the lack of interaction between HER2 and the clathrin-coated pits that induce endocytosis of other HER family members (29). However, we found that silencing of *CAV1* (encoding caveolin-1), but not *CLTA* (encoding clathrin), substantially impaired trastuzumab-induced retrograde transport of DPAGT1 (Figure 4, F and G), which suggested that the caveolae pathway, but not the clathrin pathway, was involved in the PM/ER translocation of DPAGT1. In line with this hypothesis, trastuzumab treatment drastically increased p-CAV-1^Y14^ levels in the SK-BR-3 cells (Figure 4H). Moreover, c-Src, which induces caveolin-1 tyrosine phosphorylation during endocytosis (32, 33), was also activated by trastuzumab treatment (Figure 4H). This observation was consistent with previous findings that c-Src is frequently activated in the acquired and de novo trastuzumab-resistant cells (34–36). We further observed that trastuzumab treatment resulted in enrichment of c-Src in the lipid rafts that contained DPAGT1 and caveolin-1 (Figure 4I), which was in accord with a previous report that activated c-Src translocates into the lipid rafts (37). Importantly, treatment with the c-Src inhibitor dasatinib abrogated the trastuzumab-induced retrograde transport of DPAGT1 (Figure 4J). These data provided evidence that c-Src activation is essential for the trastuzumab-induced caveolar endocytosis of DPAGT1. Although the underlying mechanism for the selection of the caveolae-mediated endocytosis pathway upon trastuzumab treatment remained unclear, it was consistent with previous reports that inhibition of several kinases would promote caveolae-mediated endocytosis (38–40).

### DPAGT1-mediated N-glycosylation protects ADAM10 from ER-associated degradation.

**Figure 5:**
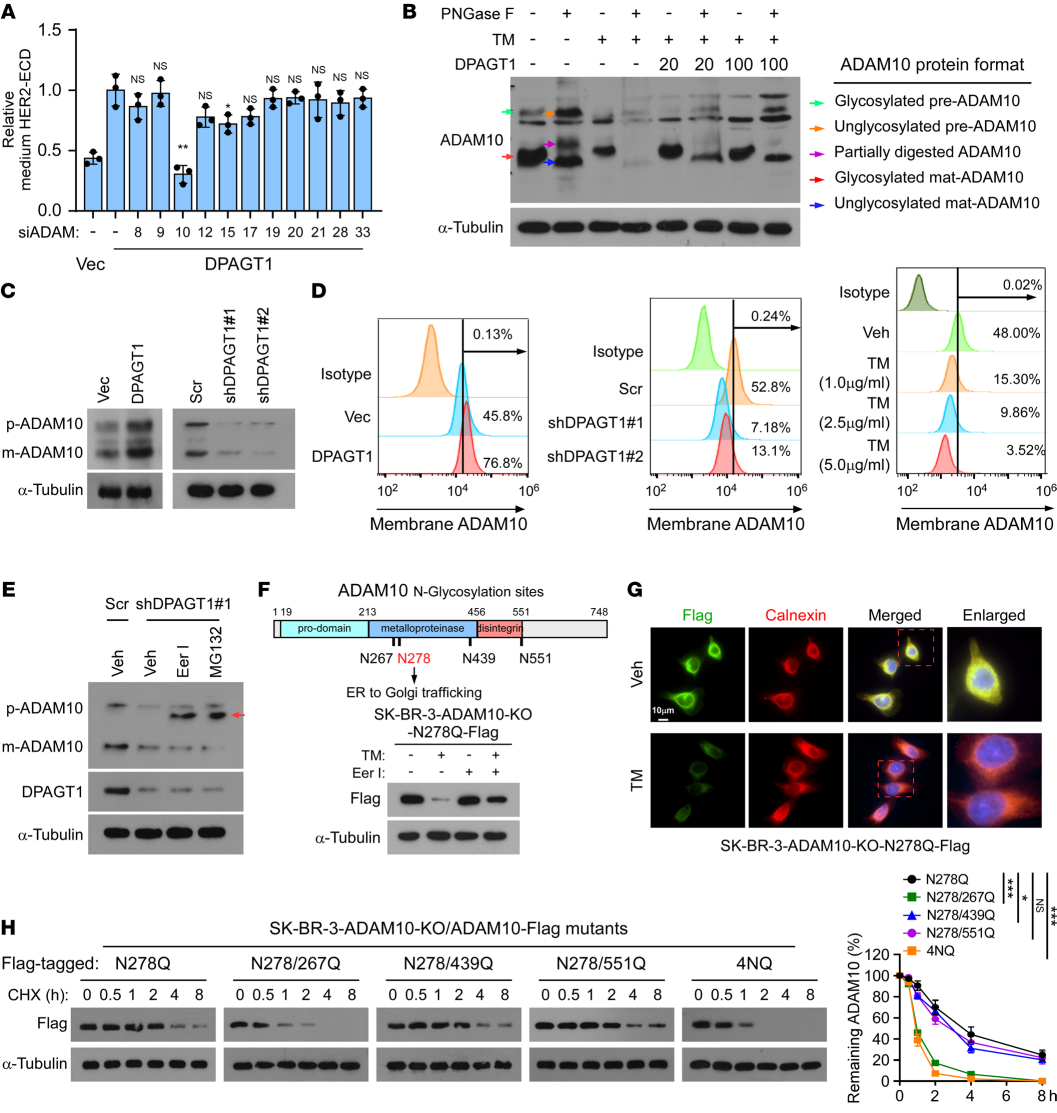
DPAGT1-mediated N-glycosylation protects ADAM10 from ER-associated degradation. (**A**) ELISA analysis of HER2-ECD level in the culture medium derived from vector control or the indicated *ADAM*-silenced SK-BR-3 cells. (**B**) IB analysis of ADAM10 expression in SK-BR-3 cells treated with or without PNGase F, tunicamycin (TM), or DPAGT1 transfection. Arrows indicated different forms of the ADAM10 protein. α-Tubulin was used as a loading control. (**C**) IB analysis of ADAM10 expression in the control or DPAGT1-dysregulated SK-BR-3 cells. α-Tubulin was used as a loading control. (**D**) Flow cytometry analysis of membrane expression of ADAM10 in the indicated SK-BR-3 cells. (**E**) IB analysis of ADAM10 and DPAGT1 expression in the indicated SK-BR-3 cells treated with vehicle, Eeyarestatin I (Eer I, 20 μM), or MG132 (10 μM). Arrow indicates the unglycosylated ADAM10 precursor. α-Tubulin was used as a loading control. (**F**) Upper: a scheme indicating the 4 N-glycosylation sites of ADAM10. Lower: IB analysis of ADAM10/N278Q-Flag expression in the SK-BR-3/ADAM10-KO cells treated with Vehicle, TM, Eer I, or TM + Eer I. (**G**) IF staining of flag-tagged ADAM10/N278Q and ER marker Calnexin in the vehicle- or TM-treated ADAM10/N278Q-Flag-transduced SK-BR-3/ADAM10-KO cells. (**H**) Cycloheximide (CHX) chase assay analysis of expression of the indicated ADAM10-Flag mutants (N278Q, 4NQ, N278/267Q, N278/439Q, and N278/551Q) in the indicated SK-BR-3-ADAM10-KO cells treated with 100 μg/ml CHX. Proteins were collected at the indicated time points and then immunoblotted with an anti-Flag antibody. Quantification of Flag-ADAM10 protein level was determined by normalization to α-tubulin protein. Data in (**A** and **H**) were plotted as the mean ± SD of biological triplicates. A 2-sided Student’s *t* test was used in **A** and 2-way ANOVA was used in **H**. *P < 0.05, **P < 0.01, ***P < 0.001.

In the ER, DPAGT1 initiates N-glycosylation of newly synthesized proteins (41). Previously, it was reported that ADAMs, disintegrin and metalloprotease family proteins, contribute to HER2 ectodomain shedding in HER2 overexpressing breast cancer cells (42). Notably, the function of ADAMs could be regulated by N-glycosylation (43–46). We then examined whether ADAMs contribute to DPAGT1-induced HER2 shedding and trastuzumab resistance. As shown in Supplemental Figure 5A and Figure 5A, among multiple ADAM family members with enzyme activity, silencing of *ADAM10* decreased the DPAGT1-induced HER2-ECD level in the culture medium, which suggested that ADAM10 might be involved in DPAGT1-mediated HER2 shedding.

N-glycosylation modulates ADAM10 functions by regulating (a) its trafficking from the ER to the Golgi apparatus, (b) protein processing in Golgi, and (c) its sheddase activity at the plasma membrane (44, 47). We first validated the impact of N-glycosylation on ADAM10. As shown in Figure 5B, treatment of SK-BR-3 lysates with Peptide:N-glycosidase F (PNGase F),an amidase that removes all N-linked oligosaccharides from glycoproteins, induced downward shifts of precursor ADAM10 (preADAM10) and mature ADAM10 (matADAM10), which suggested that both pre and matADAM10 were N-glycosylated. Meanwhile, PNGase F treatment increased the expression of preADAM10 and partially digested ADAM10 but decreased the expression of matADAM10, indicating that N-glycosylation was required for the processing and expression of ADAM10 protein. Notably, inhibition of DPAGT1 by TM dramatically reduced the levels of both glycosylated preADAM10 and matADAM10, which was enhanced by combined treatment with PNGase F, indicating that the degradation of ADAM10 might still be active in the lysates of SK-BR-3 cells. This result indicated that N-glycosylation was essential for ADAM10 expression. The different effects between PNGase F and TM on preADAM10 and matADAM10 expression might reflect the fact that PNGase F directly removes N-linked glycans from proteins in the cell lysates while TM inhibits the conjugation of N-linked glycans to proteins in live cells. Exogenous transfection of DPAGT1 slightly rescued the ADAM10 expression in the presence of TM, but no apparent dose-dependent effects were observed, suggesting that the inhibition by TM was sufficiently powerful (Figure 5B).

Consistent with the inductive effect of N-glycosylation on ADAM10 expression, overexpressing *DPAGT1* increased, while silencing *DPAGT1* reduced, the endogenous preADAM10 and matADAM10 levels in SK-BR-3 cells (Figure 5C). Meanwhile, flow cytometry analysis showed that membrane-localized ADAM10, an indicator of matADAM10, was elevated in *DPAGT1*-overexpressing cells but reduced in the *DPAGT1*-silenced or TM-treated cells (Figure 5D). Interestingly, the DPAGT1-induced upregulation of ADAM10 protein was independent of the transcription of ADAM10 (Supplemental Figure 5B). Therefore, these results suggested that DPAGT1-mediated N-glycosylation upregulated ADAM10 expression posttranscriptionally.

No endogenous unglycosylated precursor or matADAM10 protein could be detected in the SK-BR-3 cells with TM treatment or *DPAGT1* knockdown (Figure 5, B and C), suggesting that unglycosylated ADAM10 protein experienced rapid degradation in the ER, also referred to as ER-associated degradation (ERAD) (48). Consistent with this hypothesis, treatment with the ERAD inhibitor eeyarestatin I (Eer I) or the proteasome inhibitor MG132 restored the expression of preADAM10 protein, which was unglycosylated, as its molecular weight was lower than the endogenous glycosylated preADAM10 (Figure 5E). However, no restoration of matADAM10 was observed (Figure 5E). This fit well with the notion that ADAM10, without appropriate N-glycosylation modification, could not be trafficked out of the ER or processed in the Golgi apparatus (49).

ADAM10 was found to be heavily modified with N-linked glycosylation at 4 Asparagine sites (N267, N278, N439, and N551) (44). Indeed, N-glycosylation of ADAM10 could be observed in the identified peptides by the mass spectrometry (MS) analysis (Supplemental Figure 5C). We then determined which N-glycosylation site was related to ERAD of ADAM10. Since N-glycosylation at N278 is required for the trafficking of preADAM10 out of ER (44), we stably reexpressed the ADAM10/N278Q mutant in the *ADAM10*-KO SK-BR-3 cells and found that the ADAM10/N278Q mutant was trapped in the ER (Figure 5F and Supplemental Figure 5D). Although either inhibition or silencing of *DPAGT1* resulted in drastic reduction of preADAM10-N278Q in the ER, the ERAD of ADAM10/N278Q could be rescued by Eer I treatment (Figure 5, F and G and Supplemental Figure 5E). These results suggested that the other N-glycosylated sites might be indispensable for protecting preADAM10 from ERAD. As shown in Figure 5H, further mutation at N267 potently shortened the half-life of preADAM10/N278Q, which showed comparable effects to the ADAM10/4NQ (mutation of all 4 N-glycosylation sites), while further mutation at N439 or N551 showed only slight reduction of ADAM10/N278. These data indicated that glycosylation at N267 played a vital role in protecting preADAM10 from ERAD. Intriguingly, unlike the ADAM10/N278Q-N267Q and ADAM10/4NQ, ADAM10 with a single mutation at N267 (ADAM10/N267Q) expressed both preADAM10 and matADAM10, which could not be effectively restored by Eer I treatment (Supplemental Figure 5F), suggesting that the ERAD-mediated downregulation ADAM10 might be caused by the synergistic effects in N-glycosylation at multiple sites.

### Unglycosylated ADAM10 is degraded by the HRD1/SEL1L/VCP complex.

**Figure 6:**
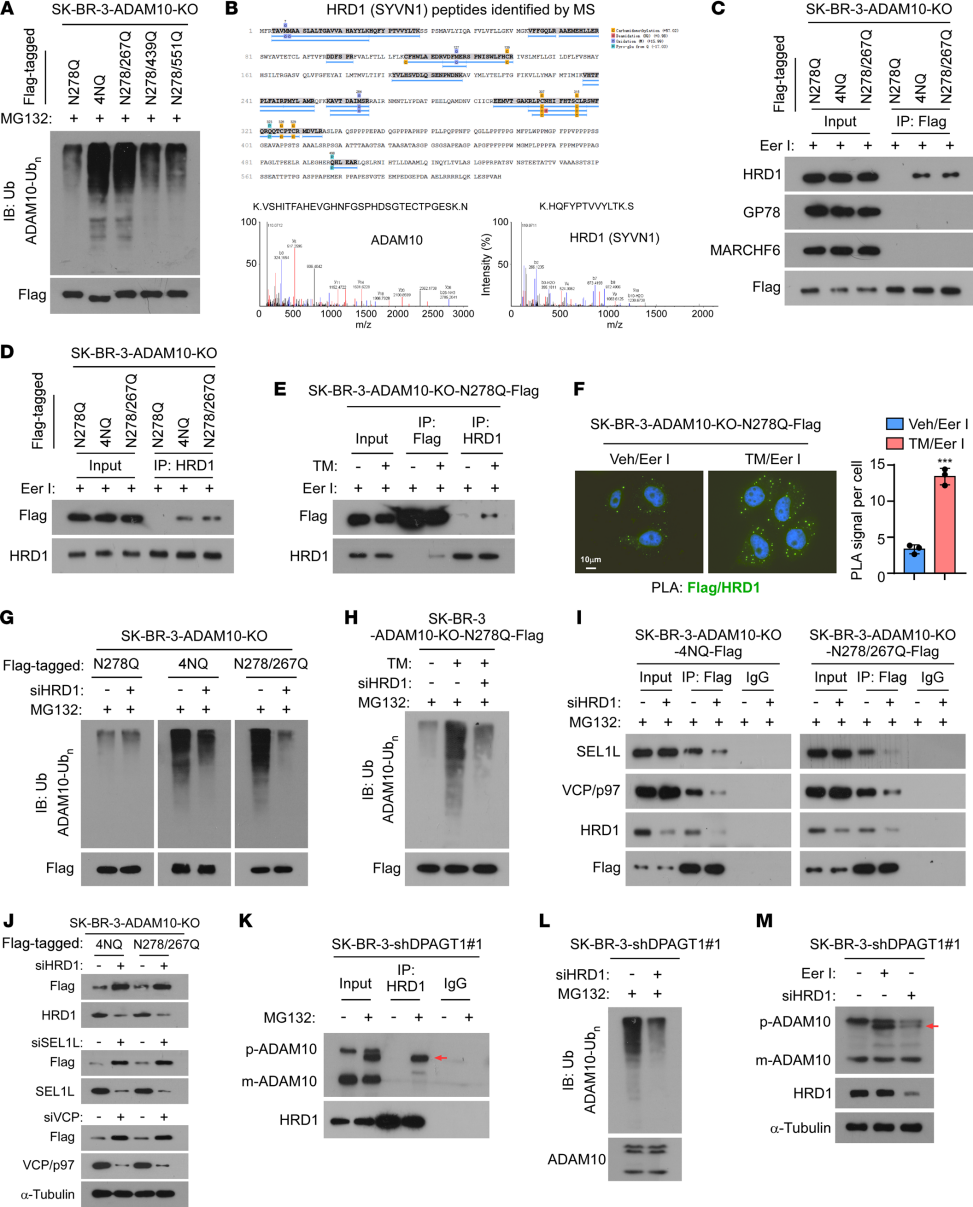
Unglycosylated ADAM10 is degraded by the HRD1/SEL1L/VCP complex. (**A**) IB analysis of poly-Ub expression of the indicated Flag-tagged ADAM10 mutants in the cells. α-Tubulin was used as a loading control. (**B**) IP/MS analysis showing ADAM10 and HRD1 (SYVN1) protein peptides in the ADAM10/4NQ-Flag complex precipitated from the Eer I-treated SK-BR-3/ADAM10-KO cells. (**C**) Co-IP assays using anti-Flag antibody were performed in the indicated cells, and IB analysis of the expression of HRD1, GP78, MARCHF6, and Flag-tagged ADAM10 mutants were shown. (**D**) IP/IB analysis of HRD1 and Flag-tagged ADAM10 mutants in the indicated SK-BR-3 cells. (**E**) IP/IB analysis of HRD1 and Flag-tagged ADAM10/N278Q in the indicated cells. (**F**) Proximity ligation assay (PLA) analysis of the interaction between ADAM10-N278Q-Flag and HRD1 upon Eer I or Eer I + TM treatment. The PLA signal was quantified by counting the foci in 5 random fields per cell. Scale bar: 10 μm. 2-sided Student’s *t* test was used. Data were plotted as the mean ± SD of biological triplicates. ***P < 0.001. (**G** and **H**) IB analysis of poly-Ub expression of the indicated Flag-tagged ADAM10 mutants in the indicated cells treated with MG132 (**G**) or MG132 + TM (**H**). (**I**) IP/IB analysis of the expression of SEL1L, VCP/p97, HRD1, and Flag-tagged ADAM10 mutants in the indicated cells. α-Tubulin was used as a loading control. (**J**) IB analysis of the expression of SEL1L, VCP/p97, HRD1, and Flag-tagged ADAM10 mutants in the indicated cells. α-Tubulin was used as a loading control. (**K**) IP/IB analysis of the levels of precursor ADAM10 (p-ADAM10), mature ADAM10 (m-ADAM10), and HRD1 in the indicated cells. Arrow indicates the unglycosylated ADAM10 precursor. (**L**) IB analysis of the poly Ubiquitination expression of ADAM10 in the control or HRD1-silenced SK-BR-3/ shDPAGT1-#1 cells. (**M**) IB analysis of expression of p-ADAM10, m-ADAM10 and HRD1 in the indicated cells. Arrow indicates the unglycosylated ADAM10 precursor. α-Tubulin was used as the loading control.

Next, we explored the mechanism underlying ERAD-mediated ADAM10 degradation. Multiple steps are involved in the process of ERAD, including that the defective protein being recognized by ERAD substrate recognition factors and specifically ubiquitinated by the ER-associated E3 ligase, and then the adaptor protein adaptor subunit of ERAD E3 ubiquitin ligase (SEL1L) links the ubiquitinated substrates and recruits the valosin-containing protein (VCP, also known as p97) complex to promote retrotranslocation by which substrate proteins are extracted from the ER and degraded by the cytosolic 26S proteasome (48, 50). Consistent with the stability of ADAM10 mutants, as shown in Figure 5H, the polyubiquitination level of ADAM10/N278-267Q and ADAM10/4NQ was markedly higher than the polyubiquitination level of ADAM10/N278Q-N439Q and ADAM10/N278Q-N551Q (Figure 6A). The ubiquitin assays using different HA-Ub constructs indicated that the polyubiquitination of ADAM10-4NQ was K48-linked but not K63-linked (Supplemental Figure 6A).

Several ER-associated E3 ligases, including synoviolin 1 (SYVN1, also known as HRD1), autocrine motility factor receptor (AMFR, also known as GP78), and membrane associated ring-CH-type finger 6 (MARCHF6), have been identified to be involved in ERAD-mediated degradation (51–53). Our high-throughput immunoprecipitation mass spectrometry (IP-MS) analysis showed that ADAM10-4NQ potentially interacted with HRD1/SYVN1 rather than GP78 or MARCHF6 (Figure 6B and Supplemental Table 3). ADAM10-4NQ peptides comprising mutations of the 4 N-glycosylation sites could not be identified by MS as they were all mutated (Supplemental Figure 6B). The IP-MS analysis was further confirmed by subsequent reciprocal co-IP assays, in which the ERAD-prone ADAM10-N278/267Q and ADAM10-4NQ mutants interacted with HRD1 but not with GP78 or MARCHF6; ADAM10-N278Q was used as a negative control (Figure 6, C and D). Furthermore, the IP assays, proximity ligation assays (PLAs) and IF staining indicated that TM treatment increased the interaction between ADAM10-N278Q and HRD1 upon Eer I treatment (Figure 6, E and F and Supplemental Figure 6C). Importantly, silencing of *HRD1* substantially reduced the polyubiquitination level of ADAM10/N278Q-267Q and ADAM10/4NQ, but had little effect on the ADAM10-N278Q polyubiquitination (Figure 6G). Moreover, the TM-induced polyubiquitination of ADAM10-N278Q was abrogated in the HRD1-silenced cells (Figure 6H). Taken together, these results demonstrate that HRD1 was responsible for the polyubiquitination of unglycosylated ADAM10 in the ER.

During the progress of ERAD-mediated protein degradation, ubiquitinated proteins are retrotranslocated out of the ER and degraded by the SEL1L and VCP/p97 complex (48). Our IP assays indicated that depletion of HRD1 repressed the binding of ADAM10-4NQ or ADAM10-N278/267Q to SEL1L and VCP/p97 (Figure 6I). Silencing *HRD1*, *SEL1L*, or *VCP* potently increased the ADAM10-4NQ and ADAM10-N278/267Q expression (Figure 6J). Therefore, these data suggest that the HRD1/SEL1L/VCP complex contributed to ERAD-mediated degradation of unglycosylated ADAM10 protein.

We further assessed the effect of HRD1 on the endogenous expression of ADAM10. Co-IP assays indicated that the HRD1 interacted with the unglycosylated preADAM10 in the SK-BR-3-shDPAGT1#1 cells treated with MG132 (Figure 6K). Silencing of *HRD1* in SK-BR-3-shDPAGT1#1 cells not only robustly decreased the level of polyubiquitinated ADAM10 but also restored the level of unglycosylated preADAM10 similar to EerI treatment (Figure 6, L–M), which further supported the notion that HRD1 played a crucial role in ERAD-mediated ADAM10 polyubiquitination.

### N-glycosylation of ADAM10 is required for DPAGT1-induced trastuzumab resistance.

**Figure 7:**
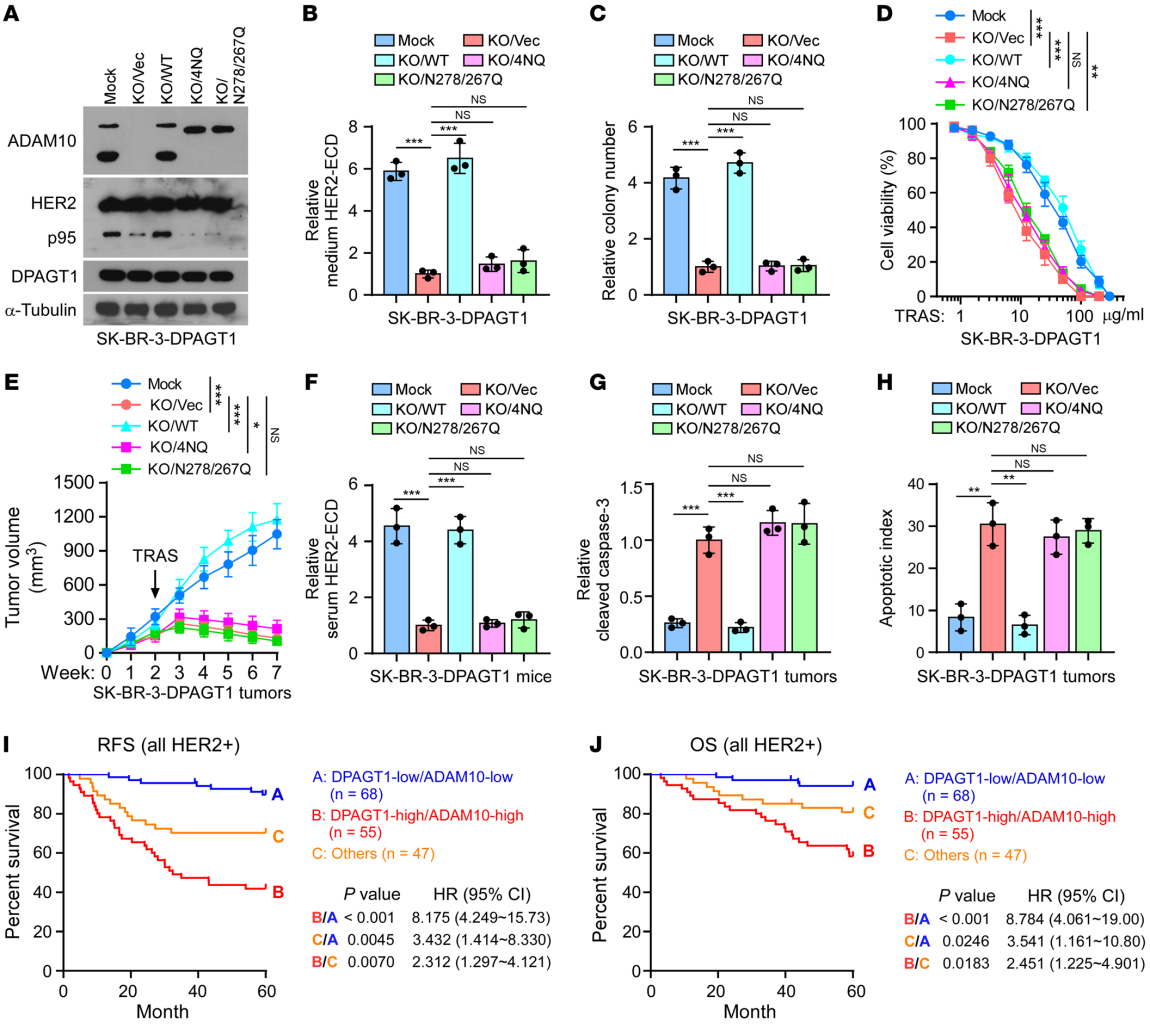
N-glycosylation of ADAM10 is required for DPAGT1-induced trastuzumab resistance. (**A**) IB analysis of the expression of ADAM10, p95, and DPAGT1 in the indicated cells. Mock represents the SK-BR-3/DPAGT1 cells. α-Tubulin was used as a loading control. (**B**) ELISA analysis of HER2-ECD level in the culture medium from the same cells in **A**. Data are presented relative to that in the KO/Vec cells. (**C**) Quantification of relative surviving colonies formed by indicated cells. Data are presented relative to that in the KO/Vec cells. (**D**) Cell viability assays analyzed the sensitivities of the indicated cells to trastuzumab. (**E**) Tumor growth curves of the xenograft tumors (*n* = 8/group) formed by the indicated cells. After 2 weeks of inoculation of the indicated cells, trastuzumab (20 mg/kg) was administered once a week for 5 weeks. Tumor volumes were measured weekly. (**F**) Relative serum HER2-ECD level in 3 mice from each group. (**G**) ELISA analysis of relative cleaved caspase-3 in indicated tumors. (**H**) The apoptotic index represented as the percentage of TUNEL^+^ cells in indicated tumors. (**I** and **J**) Kaplan-Meier analysis of RFS (**I**) and OS (**J**) curves in the patients with HER2^+^ breast cancer stratified by DPAGT1-high/ADAM10-high, DPAGT1-low/ADAM10-low, and others. Data in **B**, **C**, **D**, **G**, **H**, and **I** were plotted as the mean ± SD of biological triplicates. Data in **E** was plotted as the mean ± SD of 8 mice. Unpaired 2-sided Student’s *t* test was used in **B**, **C**, **G**, **H**, and **I**. 2-way ANOVA was used in **D** and **E**. χ^2^ test was used in **J**. *P < 0.05, **P < 0.01, ***P < 0.001.

We next assessed whether N-glycosylation of ADAM10 was required for DPAGT1-induced HER2 shedding and trastuzumab resistance. As shown in Figure 7, A–D and Supplemental Figure 7A, silencing of *ADAM10* markedly reduced the expression of intracellular p95HER2 and HER2-ECD level in the culture medium, the number of surviving colonies, and the viability of SK-BR-3-DPAGT1 cells, which could be rescued by reexpression of ADAM10-WT, but not ADAM10-4NQ or ADAM10-N278/267Q (Figure 7, A–D and Supplemental Figure 7A). The same effect of ADAM10 on HER2 shedding and trastuzumab resistance was further validated in vivo using the subcutaneous xenograft models (Figure 7E). *DPAGT1*-overexpressing SK-BR-3 tumors with *ADAM10* KO showed a sensitive response to trastuzumab treatment, as indicated by significant shrinkage of tumor volumes (Figure 7E and Supplemental Figure 7C). Meanwhile, we found that KO of *ADAM10* drastically decreased the HER2-ECD level in mouse serum, but increased the level of cleaved-caspase-3 and the apoptotic index in the SK-BR-3/DPAGT1 tumors (Figure 7, F–H and Supplemental Figure 7B). Notably, restoring ADAM10 via overexpression of ADAM10/WT restored HER2 shedding and trastuzumab resistance again, while the ADAM10/4NQ and ADAM10/N278Q-267Q overexpression did not show such effects (Figure 7, F–H and Supplemental Figure 7B). Therefore, these results indicate that N-glycosylated ADAM10 was essential for DPAGT1-induced HER2 shedding and trastuzumab resistance.

Furthermore, the clinical significance of the DPAGT1/ADAM10 axis was assessed. IHC statistical analysis revealed that high expression of ADAM10 was positively associated with shorter RFS and OS (Supplemental Figure 7D), and that ADAM10 expression correlated positively with DPAGT1 level in the 170 HER2^+^ breast cancer specimens (Supplemental Figure 7E). Importantly, patients with combined high DPAGT1 and high ADAM10 expression suffered the worse RFS and OS (Figure 7I-J). Taken together, these results suggest that the DPAGT1/ADAM10 axis is associated with poor prognosis of patients with HER2^+^ breast cancer.

### Inhibition of DPAGT1 resensitizes trastuzumab-resistant HER2^+^ breast cancer.

**Figure 8:**
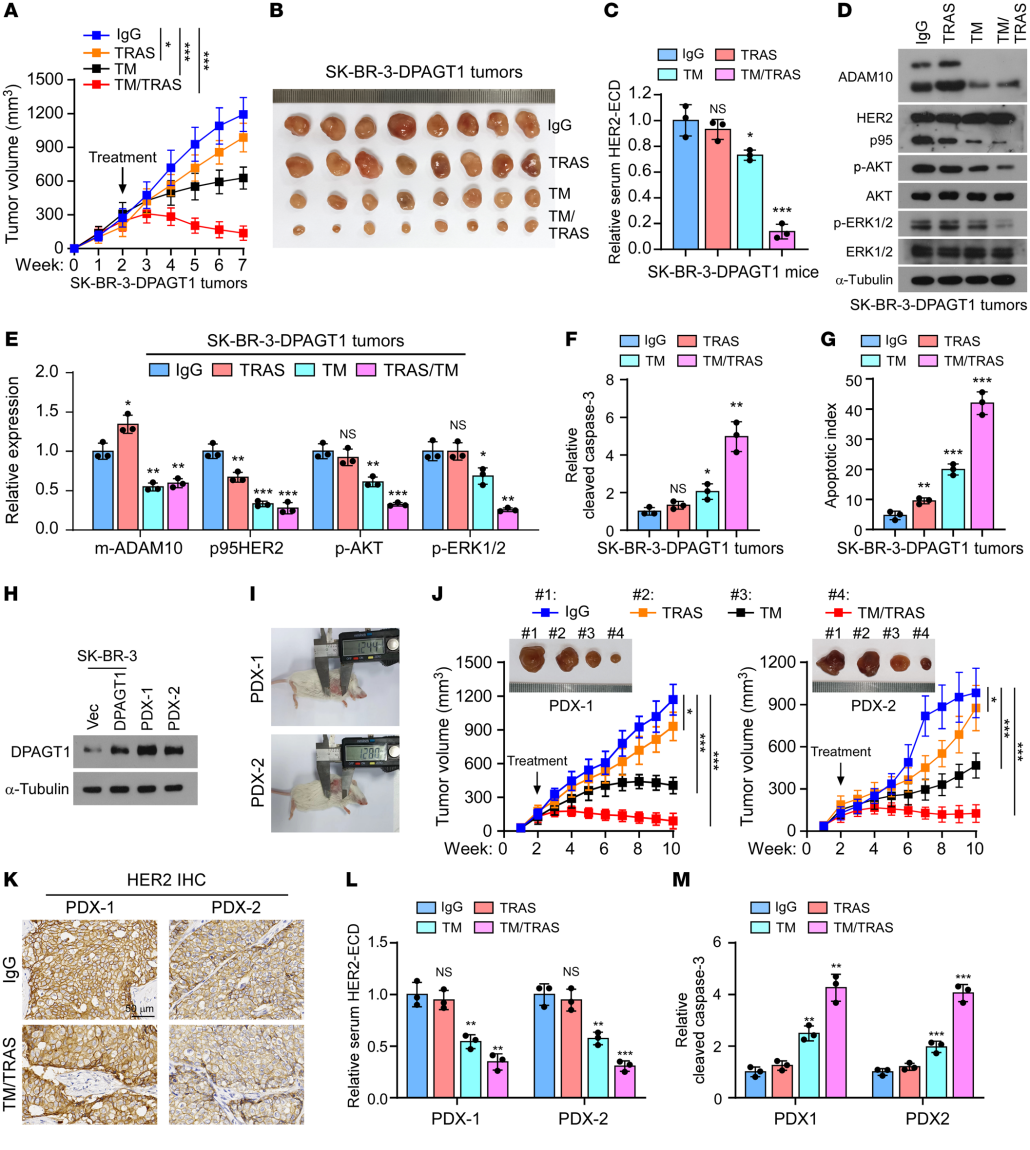
Inhibition of DPAGT1 re-sensitizes trastuzumab-resistant breast cancer. (**A** and **B**) Tumor growth curves (**A**) and representative pictures (**B**) of the xenograft tumors (*n* = 8/group) formed with SK-BR-3/DPAGT1 cells in the mice, which, after 2 weeks of inoculation of the indicated cells, were administered with IgG, trastuzumab (i.p.), TM (i.t.), or TM + trastuzumab once a week for 5 weeks. Tumor volumes were measured weekly. (**C**) ELISA analysis of the serum HER2-ECD level in the mice in each group. (**D** and **E**) IB analysis (**D**) and relative quantification (**E**) of the expression of ADAM10, HER2, p-AKT, AKT, p-ERK1/2, and ERK1/2 in the homogenates prepared from 3 random tumors in each group. (**F**) ELISA assay of the relative cleaved caspase-3 in the indicated tumors. (**G**) The apoptotic index represented as the percentage of TUNEL^+^ cells in the indicated tumors. (**H**) IB analysis of DPAGT1 expression in SK-BR-3/Vector and SK-BR-3/DPAGT1cells and in PDX-1 and -2. (**I**) Representative image of PDX-1 and PDX-2 tumor-bearing mice treated with IgG. (**J**) Representative pictures (upper) and tumor growth curves (lower) of PDX-1 and PDX-2 tumor-bearing mice with indicated treatment. *n* = 8/group. (**K**) IHC staining of HER2 in PDX-1 and PDX-2 tumor-bearing mice treated with IgG or TM + trastuzumab. Scale bar: 50 μm. (**L**) ELISA analysis of the serum HER2-ECD level in 3 mice in each group. (**M**) ELISA analysis of relative cleaved caspase-3 in the indicated PDXs. Data in **C**, **F**, **G**, **L**, and **M** were plotted as the mean ± SD of biological triplicates. Data in **A** and **J** were plotted as the mean ± SD of 8 mice. Unpaired 2-sided Student’s *t* test was used in **C**, **E**–**G**, **L**, and **M**. 2-way ANOVA was used in **A** and **J**. *P < 0.05, **P < 0.01, ***P < 0.001.

Finally, we assessed the therapeutic effect of combined TM and trastuzumab on HER2^+^ breast cancer. As shown in Supplemental Figure 8, A and B, inhibition of DPAGT1 using TM significantly enhanced the sensitivity of SK-BR-3/DPAGT1 and BT-474/DPAGT1 cells to trastuzumab therapy, as indicated by reduced cell viability and surviving colonies. The therapeutic effect of combined TM and trastuzumab on HER2^+^ breast cancer was further validated using in vivo mouse models. We first established a mouse xenograft model by s.c. injection of the trastuzumab-resistant SK-BR-3/DPAGT1 cells for 2 weeks, then treated them with either IgG, trastuzumab, TM, or trastuzumab + TM once a week (Figure 8A). To secure the anticancer effect and lessen the severe off-target toxicities by systemic administration of TM, we used an intratumoral injection (i.t.) of TM, for the purpose to secure the anticancer effect and minimize the systemic toxicities. The safety of i.t. injection of TM was tracked during the in vivo experiments. No obvious behavioral disorders in the mice and no significant alteration in mouse weight were observed during the TM treatment, indicating that this treatment was well tolerated by the mice (Supplemental Figure 8C). Remarkably, the coadministration of trastuzumab and TM dramatically shrank the volumes of SK-BR-3-DPAGT1 tumor burdens compared with TM or trastuzumab monotherapy (Figure 8, A and B). Meanwhile, we found that combined delivery of TM and trastuzumab substantially reduced the level of serum HER2-ECD, mature ADAM10, p95HER2, p-AKT, and p-ERK1/2 but increased the level of cleaved-caspase-3 expression and the apoptotic index in the SK-BR-3/DPAGT1 tumors (Figure 8, C–G and Supplemental Figure 8, D–E).

**Figure 9:**
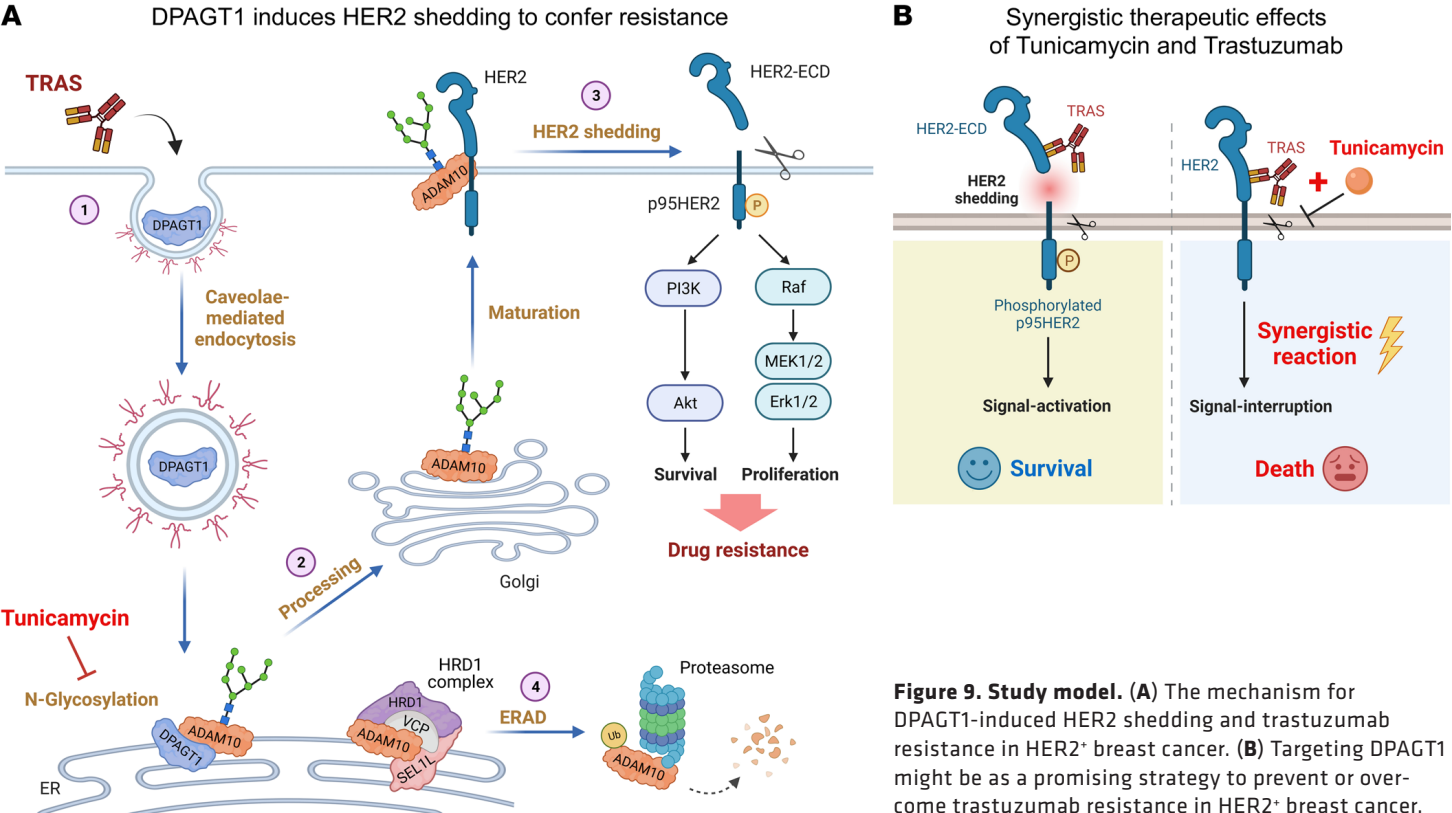
Study model. (**A**) The mechanism for DPAGT1-induced HER2 shedding and trastuzumab resistance in HER2^+^ breast cancer. (**B**) Targeting DPAGT1 might be as a promising strategy to prevent or overcome trastuzumab resistance in HER2^+^ breast cancer.

The therapeutic potential of combined TM and trastuzumab was further evaluated in patient-derived xenografts (PDXs) using the clinical trastuzumab-resistant HER2^+^ breast tumors. 2 PDXs, which expressed high levels of DPAGT1 with rapid tumor growth, were selected for the therapeutic examination (Figure 8, H and I). Similarly, intratumoral injection of TM was tolerated in the NOD/SCID mice without significant alteration in mouse weight (Supplemental Figure 8F). Although these 2 PDXs still progressed under trastuzumab treatment showing resistance to trastuzumab, combined delivery of trastuzumab and TM drastically inhibited the growth of PDX-1 and PDX-2 tumors (Figure 8J and Supplemental Figure 8G). Pathological validation showed that the 2 PDXs were still HER2^+^ after treatment (Figure 8K). Coadministration of trastuzumab and TM significantly reduced serum HER2-ECD levels but increased cleaved-caspase-3 expression in the PDXs (Figure 8, L and M). Therefore, our results demonstrated that, in HER2^+^ breast cancer, trastuzumab treatment induced PM-to-ER retrograde transportation of DPAGT1, which protected ADAM10 from ERAD via N-glycosylation, consequently resulting in robust HER2 shedding, trastuzumab resistance, and poor clinical outcomes (Figure 9A). However, combined treatment with TM and trastuzumab exerted synergistic effects on blocking HER2 signaling, suggesting that targeting DPAGT1 might be a potential strategy to reverse trastuzumab resistance (Figure 9B).

## Discussion

Shedding of the HER2 receptor results in the release of soluble HER2-ECD and the retention of membrane-associated oncogenic p95HER2 (11, 12). The levels of both fragments correlate clinically with poor prognosis and reduced therapeutic response in patients with primary or metastatic HER2^+^ breast cancer (54, 55). It has been reported that high serum levels of HER2-ECD not only result in altered pharmacokinetics of trastuzumab, possibly as its binding facilitates the rapid clearance of trastuzumab, but also leads to the neutralization of anti-HER2 antibody-mediated inhibition of cell proliferation by competing with full‑length HER2 for the anti-HER2 antibody (6, 56–58). More importantly, the remaining p95HER2 constitutively activates the downstream signaling to deliver growth and survival signals to cancer cells, promoting the activity up to 10–100-fold higher than that of the full-length HER2, thus rendering tumor cells refractory to trastuzumab (13, 14). Considering that both HER2-ECD and p95HER2 contribute to the loss of therapeutic efficacy of trastuzumab, identifying the potential upstream regulator of HER2 shedding would provide a promising targetable vulnerability that could simultaneously reduce these 2 factors. In this study, we found that upregulated DPAGT1 was correlated with poor therapeutic response to trastuzumab and worse outcome in patients with HER2^+^ breast cancer. We further demonstrated the crucial role of DPAGT1 in the regulation of HER2 shedding and sustained activation of HER2 signaling, which resulted in trastuzumab resistance. Importantly, targeting of DPAGT1 using shRNA-mediated downregulation or inhibitor-mediated deactivation drastically reversed trastuzumab resistance in HER2^+^ breast cancer tumors. Therefore, these results suggest that DPAGT1 might be a potential therapeutic target against trastuzumab resistance.

Ectodomain shedding of membrane-anchored proteins plays critical roles in activation or inactivation of multiple signal transduction pathways (9). Recently, N-glycosylation modification was found to be involved in membrane protein shedding and trastuzumab resistance. For instance, Jiang et al. found that N-glycosylation was required for the shedding of hepatic membrane serine protease Matriptase-2, a protease critical in iron homeostasis and iron-deficient anemia (59). Nagy and colleagues reported that enriched N-glycosylation of MUC4 inhibited the accessibility of trastuzumab to induce therapeutic resistance (60, 61). Herein, our data showed that upregulated glycosyltransferase DPAGT1, which induced the N-glycosylation of ADAM10 in the ER, promoted HER2 shedding and trastuzumab resistance in an enzyme-dependent manner. Importantly, DPAGT1-mediated N-glycosylation facilitated the stability and maturation of the ADAM10 sheddase, thus sustaining high levels of HER2 shedding. Therefore, these findings provide crucial evidence that N-glycosylation plays a crucial role in HER2 shedding and trastuzumab resistance.

The ADAM family is the predominant sheddase involved in ECD shedding (43). Most of the ADAM family proteins are transmembrane glycoproteins whose functions are regulated by N-glycosylation (44–46). ADAM10 has been well-characterized as a central sheddase for the shedding of many key receptors and is increased in multiple human cancers (42, 62, 63). Although N-glycosylation was found to regulate the trafficking, processing, and activation of ADAM10, the key regulator and the effect of N-glycosylation on ADAM10 remain unknown. In the current study, we demonstrated that DPAGT1 functions as a vital enzyme for N-glycosylation of ADAM10, resulting in increased stability of the ADAM10 protein by protecting ADAM10 from ERAD-mediated degradation. Consequently, N-glycosylated ADAM10-induced HER2 shedding was required for DPAGT1-promoted trastuzumab resistance. Therefore, these data decipher a crucial mechanism underlying ADAM10 N-glycosylation and upregulation in HER2^+^ breast cancer.

Resistance can be intrinsic or acquired after the tumor is exposed to certain anti-tumor therapies (64). Since p95HER2 lacks the trastuzumab recognizing epitope and is constitutively activated, the extent of HER2 shedding might represent their intrinsic capacity to antagonize trastuzumab therapy. Herein, screening of biopsies from patients who had high levels of serum HER2-ECD and poor response to trastuzumab was used to identify intrinsic promoters of HER2 shedding and trastuzumab resistance. As expected, HER2^+^ breast cancer cells and tumors with *DPAGT1* overexpression showed higher levels of HER2 shedding and were refractory to trastuzumab. High expression of DPAGT1 in the primary HER2^+^ breast tumors was associated with poor prognosis in patients, indicating that DPAGT1 intrinsically contributed to trastuzumab resistance. Notably, HER2 shedding could be repressed by trastuzumab (27), indicating that an adaptive mechanism might be required to sustain high-level HER2 shedding for resistance. Although the DPAGT1 expression was not affected by trastuzumab, our findings revealed that DPAGT1 underwent retrograde transport from the PM to the ER upon trastuzumab treatment, where DPAGT1 initiated the N-glycosylation of ADAM10 to facilitate its expression and maturation. Thus, the retrograde transport of DPAGT1 might increase the sheddase activity of ADAM10, thereby enhancing HER2 shedding and antagonizing trastuzumab therapy. Therefore, besides its overexpression in cancer, heterogeneity in the subcellular location of DPAGT1 protein might also contribute to the acquisition of drug resistance.

In the current study, TM, as a potent inhibitor of glycosylation (41), was applied to inhibit DPAGT1 activity. Although the anticancer effects of TM in various cancers have been reported for decades, TM treatment also displays severe toxicities in several types of normal cells, such as neurons and hematopoietic cells, which retarded its clinical applications (65, 66). Notably, multiple laboratories are striving to develop TM analogues with low toxicity and strong tumor-killing activity. For instance, Michio Kurosu’s (University of Tennessee, Memphis, Tennessee, USA) group has prepared new compounds, including aminouridyl phenoxypiperidinbenzyl butanamide (APPB) and capuramycin phenoxypiperidinylbenzylamide analogue (CPPB), as novel inhibitors of DPAGT1 (67–70). Importantly, APPB at a low concentration has already displayed selective inhibition in multiple solid tumors with limited toxicities toward normal cells (67–69), and CPPB treatment alone could suppress the migration of cancer cells and showed synergistic effects with chemotherapy in pancreatic cancer (70). Prominently, both APPB and CPPB exhibited hemolytic activity that were both tolerable in vivo. Thus, further exploration of the anticancer effect of APPB, CPPB, or other TM-derived analogs in trastuzumab-resistant HER2^+^ breast cancer is warranted.

In addition to HER2^+^ breast cancer, trastuzumab is now clinically applicable in HER2^+^ gastric cancer and other human HER2^+^ cancer types (26). Elevated serum HER2-ECD levels were also observed in advanced HER2^+^ gastric cancer, which led to unsatisfactory anti-HER2 therapy effect in these patients (15, 16). Intriguingly, analysis of the TCGA data showed that *DPAGT1* expression was increased in multiple types of HER2-overexpressing human cancer (26), including breast cancer, gastric cancer, colorectal cancer, and uterine corpus endometrioid carcinoma. Therefore, it would be of great significance to further investigate the effect of targeting of DPAGT1 on HER2 shedding and trastuzumab resistance in HER2^+^ pancancer types, which might represent a promising strategy against trastuzumab-resistant HER2^+^ cancer.

## Methods

### Patient specimens.

Serum and tumor biopsies were collected from 61 patients with HER2^+^ breast cancer before they received neoadjuvant trastuzumab therapy. Serum was subjected to HER2-ECD examination by ELISA, while the tumor biopsies were preserved with RNAlater Solution at –80°C until use. This study also included 170 HER2^+^ breast cancer specimens, which were clinically and histopathologically diagnosed at the Sun Yat-sen University Cancer Center. The specimens were derived from 94 patients who received HER2-targeted therapy and 76 patients who never received such therapy. The clinicopathological characteristics are summarized in Supplemental Table 1.

### IHC staining of patient specimens.

IHC staining was performed on the 170 HER2^+^ breast cancer tissue sections using anti-DPAGT1 (Sigma-Aldrich; HPA053878) and anti-ADAM10 (Sigma-Aldrich; SAB3500181) antibodies. The IHC scoring was performed in the 170 HER2^+^ breast cancer slides using 5 random 200 × tumor fields per slide by 2 independent pathologists who were blinded to the clinical outcomes. The staining of DPAGT1 and ADAM10 were graded with 4 scores, strong 3+, moderate 2+, weak 1+, and negative 0. Specimens with scores of 3+ or 2+ were defined as having high expression, while those with scores of 1+ or 0 were defined as having low expression. Slides considered controversial in DPAGT1 or ADAM10 expression were assessed and determined by a third pathologist. Details of the IHC method are provided in the Supplemental information.

### Xenograft tumor models.

Immune-deficient BALB/c-nude mice (Female, 4–5 weeks old) and NOD-SCID (Female, 4–5 weeks old) obtained from Gempharmatech Co., Ltd. were used for examination of the in situ growth of SK-BR-3 tumors and the s.c. growth of patient-derived xenograft (PDXs), respectively. Mice were housed in barrier facilities on a 12 hour light/dark cycle. In the in situ models, SK-BR-3 cells (2 × 10^6^) expressing vector, DPAGT1, or DPAGT1-N185A were orthotopically injected into the mammary fat pads of BALB/c nude mice (*n* = 8/group). Drugs were administered 2 weeks after tumor inoculation. Trastuzumab (20 mg/kg) was injected i.p., while TM (6 μg) was administered i.t. weekly for 5 weeks. Tumor volumes were measured weekly. For the relapse model (*n* = 15/group), all SK-BR-3 cells were stably transduced with the luciferase gene. Tumorectomy was performed 2 weeks after cell injection, and tumor resection was confirmed a week later by failed detection of luciferase signal using an In vivo Imagining System (IVIS, Caliper Life Sciences). Trastuzumab treatment was subsequently administered for 8 weeks (i.p., 20 mg/kg, once a week), and tumor relapse was traced by the luciferase signal.

In the PDX model, fresh HER2^+^ breast tumors derived from patients that had high serum HER2-ECD and poor response to trastuzumab, were divided into small pieces (3–5 mm) and transplanted s.c. into the left armpit of anesthetized NOD-SCID mice. When the tumors reached 1.0–1.5 cm in diameter, they were implanted into new mice and passaged a minimum of 3 times to establish model stability. Treatment was started at a tumor size of approximately 200 mm^3^. Groups of 8 mice were randomized to receive either IgG (i.p., 20 mg/kg) as control, trastuzumab (i.p., 20 mg/kg), TM (i.t., 6 μg), or trastuzumab (i.p., 20 mg/kg) combined with TM (i.t., 6 μg), once a week for 8 weeks. Serum was gathered from 3 random mice in each group before euthanization and subjected to HER2-ECD detection. The formed tumors were then used for validation of HER2 shedding, HER2 signaling, cleaved caspase-3, proliferation index, or apoptotic index.

Additional methodological information is provided in Supplemental Materials and Methods.

### Statistics.

Statistical analyses were performed using the SPSS version 19.0 statistical software package (IBM Corp.). Statistical tests for data analysis included the log-rank test, χ^2^ test, 2-tailed Student’s *t* test, and 2-way ANOVA. Multivariate statistical analysis was performed using a Cox regression model. *P* < 0.05 was considered statistically significant.

### Study approval.

Ethics approval (#B2022-459-01) and prior patient consent were obtained from the Institutional Research Ethics Committee of Sun Yat-sen University Cancer Center to use the clinical specimens for research purposes. Animal experimental procedures were approved by the IACUC of Sun Yat-sen University (No. L102042020000C). The study conformed to the principles set out in the Declaration of Helsinki.

### Data availability.

The RNA-Seq data of the 27 HER2^+^ breast cancer biopsies have been deposited in the National Center for Biotechnology Information Sequence Read Archive (SRA) database (PRJNA860770). The gene expression profile of each sample was analyzed quantitatively using the Cuffdiff suite from Cufflinks (v2.2.1) (71). The mass spectrometry data regarding the peptides and counts of ADAM10-4NQ-Flag–interacting proteins have been deposited to the ProteomeXchange Consortium (http://proteomecentral.proteomexchange.org) via the iProX partner repository with the data set identifier PXD035378.

## Author contributions

MY, YL, LK, SH, and LH carried out most of the experimental work; they collected and analyzed the data. MY, YL, SM, and XH conducted the RNA-Seq, qRT-PCR, ELISA, IF staining, and PLA assays. SH, LK, PL, YX, DS, and X Lin collected tissues and patient information and conducted IHC and survival analysis. MY, LH, SM, XC, and X Lu conducted the immunoblotting analysis, plasmid constructions, and IP assays. MY, SH, YL, and BC conducted animal studies. YO, LH, and BC conducted cell culture. MY, SM, YL, LH, and X Lu performed the in vitro studies. JL, CL, and LS raised the concept, design the experiments, wrote the manuscript, and supervised the project. The order of the co–first authors was assigned based on their efforts and contributions to the study. All author read and approved the final manuscript.

## Supplementary Material

Supplemental data

Supplemental table 3

Supporting data values

## Version 1 07/17/2023

Electronic publication
