# Supplementary material for: Inhibition of DPAGT1 suppresses HER2 shedding and trastuzumab resistance in human breast cancer
Source: J Clin Invest. 2023 Jul 17;133(14):e164428. doi: 10.1172/JCI164428 (PMC10348774; doi:10.1172/JCI164428)
Supplement: Supplemental data [file jci-133-164428-s079.pdf]

SUPPLEMENTARY INFORMATION

Supplementary Materials and Methods

Cells

The human HER2+ breast cancer cell lines SK-BR-3 and BT-474 were obtained from the American Type Culture Collection (ATCC, Manassas, VA, USA). Cell lines were authenticated by short tandem repeat (STR) fingerprinting at Medicine Lab of Forensic Medicine Department of Sun Yat-sen University. SK-BR-3 cells were grown in McCoy's 5A medium and BT-474 cells were grown in DMEM medium. Trastuzumab-resistant SK-BR-3 and BT-474 cell lines (SK-BR-3-TR and BT-474-TR) were established by exposure to increasing concentrations of trastuzumab. Briefly, SK-BR-3 or BT-474 cells were exposed to an initial trastuzumab concentration of 5 μg/ml for two days, and then kept with a drug-free culture medium until the next mitotic phase. Resistant cells were finally maintained with trastuzumab (100 μg/ml).

Quantitative detection of HER2-ECD

Quantitative detection of HER2-ECD expression in the serum of patients with HER2+ breast cancer, or tumor-bearing mice serum, or cell culture medium was examined by a 96-well enzyme-linked immunosorbent assay (ELISA, R&D Systems, #DHER20) based upon a Sandwich assay principle according to the manufacturer’s instructions. Appropriate sample dilutions were made if needed. Each sample, standard, and control were assayed in triplicate. The optical density (OD value) of each well was determined using a microplate reader set to 450 nm.

Chemicals

Trastuzumab (Herceptin®, Roche, Basel, Switzerland) contained 440 mg lyophilized trastuzumab powder per bottle. For Pertuzumab (Perjeta®, Roche), each vial contained 14 ml of Pertuzumab at a concentration of 30 mg/ml..Tunicamycin (T7765, Sigma-Aldrich, St.

Louis, MO, USA) was dissolved in DMSO for subsequent experiments. The proteasome inhibitor MG132 (M8699, Sigma-Aldrich) was used at 10 μM in the indicated experiments.

Glycosylation analysis of ADAM10 *in vitro*.

To confirm the N-glycosylation of ADAM10 protein, the cell lysates were treated with recombinant PNGase F (P0704S, New England BioLabs, Ipswich, MA, USA) as described by the manufacturer. Briefly, we mixed 1-20 μg of glycoprotein, 1 μl of Glycoprotein Denaturing Buffer (10×), and H_2O (if necessary) to make a 10 μl total reaction volume. Then denature glycoprotein by heating reaction at 100°C for 10 minutes. Add 1 μl PNGase F and incubate the reaction at 37°C for 1 hour. Assessing the extent of deglycosylation by mobility shifts on SDS-PAGE gels. ADAM10 protein was then measured using the indicated antibody.

Immunohistochemistry (IHC)

In this study, IHC staining was carried out in 170 human HER2+ breast cancer tissues and mice tumors. In brief, paraffin-embedded specimens were cut into 4-μm sections and baked at 65°C for 30 min. The sections were deparaffinized with xylenes and rehydrated. Sections were then submerged into EDTA antigenic retrieval buffer and microwaved for antigenic retrieval. Samples were treated with 3% hydrogen peroxide in methanol to quench the endogenous peroxidase activity, followed by incubation with 1% bovine serum albumin to block nonspecific binding, and then incubated with primary antibodies overnight at 4°C. After washing, the tissue sections were treated with biotinylated anti-rabbit secondary antibody, followed by further incubation with streptavidin-horseradish peroxidase complex (Zsbio, Beijing, China). Finally, the sections were immersed in 3-amino-9-ethyl carbazole and counterstained with 10% Mayer's hematoxylin, dehydrated, and mounted in Crystal Mount. Primary antibodies used in the IHC staining include anti-DPAGT1 (Sigma-Aldrich, HPA053878), anti-ADAM10 (Sigma-Aldrich, SAB3500181), anti-Ki67 (CST, Danvers, MA, USA, #12202), anti-p-ERK1/2 (CST, #4370), anti-p-AKT (CST, #4060). For negative

controls, the primary antibody was replaced with normal rabbit serum at 4°C overnight preceding the immunohistochemical staining procedure.

Plasmids, retroviral infection and establishment of stable cell lines

Human DPAGT1 and ADAM10 coding sequence were amplified by the polymerase chain reaction and subcloned into a pLVX-retro-puro and pLVX-retro-hygro vector. ADAM10 mutants were constructed based on the ADAM10-wild-type (wt) and tagged with the Flag peptide sequence. To silence endogenous DPAGT1, two short hairpin RNA (shRNA) oligonucleotides were cloned into the pSuper-retro-neo vector, respectively. The oligonucleotide sequences of shRNAs were provided below.

The stable cells were generated from cell pools by retroviral infection using the pLVX-retro-puro and pLVX-retro-hygro for DPAGT1 or ADAM10 overexpression, and p-Super-retro-neo for DPAGT1 silencing. Briefly, the retroviral vectors were co-transfected with packaging plasmid into 293T cells. The supernatant containing the virus was collected, and viral infections were done serially for 3 days. Stable cell lines were selected with 0.5 μg/ml puromycin, 25 μg/ml hygromycin, or 250 μg/ml G418, respectively. ADAM10 knockout (KO) SK-BR-3 cells were established by CRISPR/Cas9 system. ADAM10-KO clones were isolated by single-cell dilution cloning from the positive polyclonal sgRNA-transduced populations and were identified by immunoblotting.

Immunoblotting (IB) analysis of HER2-ECD and p95HER2

The indicated cells were lysed using 500 ml cold lysis buffer (50 mM Tris/HCl, 1 %TritonX-100 pH 7.4, 1 % sodium deoxycholate, 0.1 % SDS, 0.15 % NaCl, 1 mM EDTA, 1 mM sodium orthovanadate) at 4 °C. The culture medium of SK-BR-3 and BT-474 were collected and concentrated 20-fold by freeze-drying. The protein concentration of the samples was determined using a BCA Protein Assay Kit (Beyotime, Jiangsu, China). Equal amounts

of protein (30 μg) were subjected to SDS electrophoresis and blotted onto the PVDF membranes. Proteins were incubated with the following antibodies: (i) anti-HER2 (29D8) (CST), against intracellular domain (ICD); (ii) HER2 (D8F12) (CST), against extracellular domain (ECD) overnight at 4 °C, and then with the appropriate secondary antibodies. Detection of protein bands was performed using the ECL kit (Bio-Rad Laboratories, Hercules, CA, USA). α-Tubulin was used as a loading control for lysate proteins, and Albumin was used as a loading control for medium proteins.

Cell viability

Cell viability was examined using the 3-(4, 5-Dimethyl-2-thiazolyl)-2, 5-diphenyl-2H-tetrazolium bromide (MTT) assay. Approximate 2.5×10^3 cells were plated in 96-well plates. MTT assay solution (#11465007001, Cell Proliferation Kit I Roche) was added to each well and incubated for 2 h. The medium was then aspirated off and cells were resuspended in 200 μL of DMSO. Absorbance at 560 nm was measured, with the background at 670 nm subtracted. The percentage survival of drug-treated cells versus vehicle-treated cells was calculated.

Polyubiquitination analysis

To analyze the polyubiquitination of ADAM10, the indicated cells was treated with 10 μM of the proteasome inhibitor MG132 for 6 h and then washed with PBS, pelleted, and lysed in HEPES buffer (20 mM HEPES, pH 7.2, 50 mM NaCl, 1 mM NaF, 0.5% Triton X-100) plus 0.1% SDS, 10 μM MG132 and protease-inhibitor cocktail. The lysates were centrifuged to obtain cytosolic proteins and incubated with anti-Flag (CST, #14793) overnight. The lysates were then pulldown with agarose beads. The beads were washed six times with HEPES buffer and then eluted with 200 μl of 1 M glycine (pH 3.0). The proteins were boiled in SDS–PAGE sample buffer for 5 min and analyzed by IB analysis with an anti-Ub antibody (CST, #3936).

Immunoprecipitation (IP) assay

Cell lysates were prepared from the indicated cells using lysis buffer (150 mM NaCl, 10 mM HEPES, pH 7.4, 1% NP-40), and then incubated with the indicated primary antibodies and protein G-conjugated agarose (Millipore, Billerica, MA, USA) overnight at 4°C. Beads containing affinity-bound proteins were washed 6 times by wash buffer (150 mM NaCl, 10 mM HEPES, pH 7.4, 0.1% NP-40), followed by elution using 1 M glycine (pH 3.0). Elutes were subjected to mass spectrometry or western blotting analysis. In this study, antibodies used for the IP assay including anti-Flag antibody (F1804, mouse, Sigma-Aldrich; or #14793, CST, rabbit), anti-HRD1 antibody (#14733, rabbit, CST; or SAB4200423, mouse, Sigma-Aldrich). Interacting proteins were examined with primary antibodies derived from biological hosts different from those used in IP to avoid high background. The mass spectrometry data about the peptides and counts of ADAM10-4NQ-Flag interacting proteins have been deposited to the ProteomeXchange Consortium (http://proteomecentral.proteomexchange.org) via the iProX partner repository with the dataset identifier PXD035378.

Proximity ligation assay

Proximity ligation assay was performed using a Rabbit PLUS and Mouse MINUS Duolink *in situ* PLA kit (Sigma-Aldrich) according to the manufacturer's protocol. Briefly, the indicated cells on coverslips were fixed in 3.7% formaldehyde in PBS for 15 min at room temperature and then washed with TBS and blocked for 2 h with 1% BSA in TBST in a humidified chamber at room temperature. The cell coverslips were incubated overnight at 4 °C with anti-Flag (Sigma-Aldrich, F1804, mouse) and anti-HRD1 (CST, #14733, rabbit) antibodies. After washing with TBST, proximity ligation was performed using the PLA kit (Sigma-Aldrich). Cells were further counterstained with DAPI (Sigma-Aldrich) to visualize the nuclei. PLA signals were detected using an Olympus BX51 microscope (Olympus, Tokyo,

Japan) under ×40 objectives and analyzed using a macro of ImageJ software (2.0.0; NIH, Bethesda, MD, USA). The PLA signal was quantified by counting the foci per cell from five random fields.

Immunofluorescence staining

The indicated cells (5×10^4) were plated on coverslips and received indicated treatments. The cells were washed three times with PBS and treated with 1% Triton X-100. Next, cells were stained with primary antibodies overnight at 4°C according to the manufacturer's instructions. The antibodies include anti-DPAGT1 (Sigma-Aldrich, SAB3500683, rabbit), anti-Calnexin (Sigma-Aldrich, C7617, mouse), and anti-Flag (CST, #14793, rabbit). After washing three times with PBS, the cells were incubated with rhodamine-conjugated goat anti-rabbit or anti-mouse antibody (CST, 1:100) at 37°C for 1 hour. Cells were counterstained with DAPI (Sigma-Aldrich) to visualize the nuclei.

Membrane ADAM10 expression by flow cytometry analysis

To determine the membrane levels of ADAM10, the indicated cells were incubated with phycoerythrin (PE) isotype control mouse IgG or mouse ADAM10 Ectodomain PE-conjugated Antibody (R&D systems, FAB946P). After incubation, quantification was performed with a Beckman FC500 flow cytometer and analyzed with FCS Express software (De Novo Software, Pasadena, CA, USA).

Flow cytometric quantitation of membrane receptors

The expression of membrane HER2, HER3, and EGFR were measured by flow cytometry in the indicated cells after IgG or antibodies treatment. In brief, the harvested cells were resuspended in 1X PBS (1×10^4 cells/ml) and incubated with antibodies against HER2 (BD Horizon BV650-conjugated anti-HER-2 antibody, #747614, BD Biosciences, San Jose, CA, USA), HER3 (BD Horizon BV480-conjugated anti-HER-3 antibody, #751797, BD

Biosciences), and EGFR (PE/Cy7-conjugated anti-EGFR antibody, ab239309, Abcam, Cambridge, MA, USA) or the relevant isotype control at room temperature for 30 min. Following the wash steps, the cells were analyzed with a flow cytometer (CytoFLEX, Beckman,, Brea, CA, USA). A minimum of three independent measurements was run for each antibody and group. Histograms showed the input cells and the gated data indicated the levels of fluorescence. The percentages of positive cells were obtained using FlowJo v10 software.

Colony formation assay

Approximately 1×10^3 cells were plated in 6-well plates. Ten days later, the colonies were fixed, stained with crystal violet, and counted.

Terminal deoxynulceotidyl transferase nick-end-labeling (TUNEL) assay

The TUNEL assay was performed using DeadEnd™ Fluorometric TUNEL System (#G3250, Promega, Madison, WI, USA) according to the manufacturer’s protocol. Briefly, the tumor sections were deparaffinized, rehydrated, heated in a microwave oven for 1 min, and then immersed in Tris-HCL, 0.1 M PH7.5, containing 3% BSA and 20% normal bovine serum for 30 min at room temperature. TUNEL reaction mixture was then added to the sections for 60 min at 37 °C. In each case, 500–1000 cells were counted and the mean apoptotic index was calculated.

Primers and oligonucleotides used in the present study

Primers for qRT-PCR

DPAGT1, forward primer, 5'- GCGGATGATGTACTGAATCTGC -3'; reverse primer, 5'- ACAATGGTCGTGTTGCCAAAG -3';

ADAM10, forward primer, 5'- ATGGGAGGTCAGTATGGGAATC -3'; reverse primer, 5'- ACTGCTCTTTTGGCACGCT -3';

GAPDH, forward primer, 5'-GTCTCCTCTGACTTCAACAGCG-3'; reverse primer, 5'-ACCACCCTGTTGCTGTAGCCAA-3'.

Gene silencing oligonucleotides

shDPAGT1#1: 5'- GCATGATCTTCCTGGGCTTTG -3'

shDPAGT1#2: 5'- GCAAGACCATGCTACTATTCT -3'

siCLTA#1: 5'- CCGGATGCTGTTGATGGAGTAATGA-3'

siCLTA#2: 5'- CAGCTATTTCACAAGTGGATCGATT-3'

siCAV1#1: 5'- CTTTGAAGCTGTTGGGAAA -3'

siCAV1#2: 5'- GACGTGGTCAAGATTGACTTT -3'

siHRD1#1: 5'- GGCTTTGAGTATGCCATCCTGATGA -3'

siHRD1#2: 5'- CCTACTACCTCAAACACCAGTTCTA -3'

siSEL1L#1: 5'- GAAACCAGCTTTGACCGCCATTGAA -3'

siSEL1L#2: 5'- CAGACTGTGGTGTGCTACAACCTAT -3'

siVCP#1: 5'- CCAAGATGGATGAATTGCAGTTGTT -3'.

siVCP#2: 5'- GAGACTGGAGCCTTCTTCTTCTTGA -3'.

siDPAGT1#1: 5'- CCTCATGGTCTATTTCACCAACTTT -3'.

siDPAGT1#2: 5'- TCAGCAAGACCATGCTACTATTCTT -3'.

siMPP2#1: 5'- GGAAGATTTGATGTGGGTCGCTATG -3'.

siMPP2#2: 5'- CAACCTGTATGGCACACGTAT -3'.

siNEURL1#1: 5'- GAGTTTGCCAATGAGGGCAACATCA -3'.

siNEURL1#2: 5'- GACTCGGCTGTTATGCTGTTCTTCA -3'.

siCRABP1#1: 5'- CCACTGCACGCAAACTCTTCTTGAA -3'.

siCRABP1#2: 5'- GCACCAGAATTTATGTCCGAGAGTG -3'.

siRBM24#1: 5'- CAACTTCATCCAGCCCTTATACAAA -3'.

siRBM24#2: 5'-AACCAAGGATCATGCAACCAGGTTT -3'.

siSBSN#1: 5'- CAACCATGGTATTGGACAA -3'.

siSBSN#2: 5'- GATGGCATCAACAGTGGAATC -3'.

siPI3#1: 5'- AGATAAAGTCAAAGCGCAA -3'.

siPI3#2: 5'- CGTGTTCCATTCAATGGACAA -3'.

siREPS2#1: 5'- CCGGTACGGATAGAGAGTATTAAAT -3'.

siREPS2#2: 5'- TCGGATGGAGAAGACATCTGTTAAA -3'.

siCYP2T1P#1: 5'- GCGTAAGGTTAGAATGGAGGTTCAT -3'.

siCYP2T1P#2: 5'- CCAATGTGTGGATAGGGCACTTGGA -3'.

siCDH22#1: 5'- CATCATCAAGGTGCAGGACATCAAT -3'.

siCDH22#2: 5'- ACACAGACATGACTTACCACCTTAA -3'.

siNTRK3#1: 5'- TCGTCATGATCAACAAATA -3'.

siNTRK3#2: 5'- CCAATCTACCTGGACATTCTT -3'.

siCYP2B7P#1: 5'- GATAGAGGAACTTCGGAAA -3'.

siCYP2B7P#2: 5'- CCACCACCATCTAGTTCCAAACATT -3'.

siZNF239#1: 5'- CCATAATAACTGTGGGAAA -3'.

siZNF239#2: 5'- CCTTCAAGTTAAACTGGTGTCTGAT -3'.

siSTON2#1: 5'- CGTCAAAGGGAATGAAATA -3'.

siSTON2#2: 5'- CCTAGCTTTGGATGTTCGTAT -3'.

siEGFR#1: 5'-CACAGTGGAGCGAATTCCTTTGGAA -3'.

siEGFR#2: 5'-CGCAAAGTGTGTAACGGAATAGGTA -3'.

siHER3#1: 5'- GGCCATGAATGAATTCTCTACTCTA-3'.

siHER3#2: 5'-CCATCTTCGTCATGTTGAACTATAA-3'.

Antibodies used in the present study

Name	Catalogue No.	Source
**IHC**		
anti-DPAGT1	HPA053878	Sigma-Aldrich
anti-ADAM10	SAB3500181	Sigma-Aldrich
anti-Ki67	#12202	CST
anti-p-ERK1/2	#4370	CST
anti-p-AKT	#4060	CST
anti-HER-2/neu	05278368001	Roche
**WB/IP**		
anti-DPAGT1	SAB3500683	Sigma-Aldrich
anti-HER2 (ECD)	#4290	CST
anti-HER2 (ICD)	#2165	CST
anti-p-AKT	#4060	CST
anti-AKT	#9272	CST
anti-p-ERK1/2	#4370	CST
anti-ERK1/2	#4695	CST
anti-α-Tubulin	T9026	Sigma-Aldrich
anti-Albumin	#66271	CST
anti-PMCA1	PA1-914	Invitrogen
anti-Calnexin	C7617	Sigma-Aldrich
anti-Caveolin-1	16447-1-AP	proteintech
anti-ADAM10	#14194	CST
anti-Flag	F1804	Sigma-Aldrich
anti-Flag	#14793	CST
anti-Ubiquitin	#3936	CST
anti-HRD1	#14773	CST
anti-HRD1	SAB4200423	Sigma-Aldrich
anti-GP78	#9590	CST
anti-MARCHF6	PA5-103816	Invitrogen
anti-SEL1L	S3699	Sigma-Aldrich
anti-VCP	10736-1-AP	proteintech

Anti-p-CAV-1^{Y14}	#3251	CST
Anti-c-Src	#2108	CST
Anti-p-c-Src^{Y416}	#2101	CST
Anti-HER3	#12708	CST
Anti-EGFR	#54359	CST
anti-DPAGT1	SAB3500683	Sigma-Aldrich
anti-Calnexin	C7617	Sigma-Aldrich
anti-Flag	#14793	CST
anti-Flag	F1804	Sigma-Aldrich
anti-HRD1	#14773	CST
**FACS**		
ADAM10 Ectodomain PE-conjugated Antibody	FAB946P	R&D systems
BV650-conjugated anti-HER-2 antibody	#747614	BD Biosciences
BV480-conjugated anti-HER-3 antibody	#751797	BD Biosciences
PE/Cy7-conjugated anti-EGFR antibody	ab239309	Abcam

Supplementary Table 1. Clinicopathological characteristics of 170 HER2+ breast cancer specimens

**Parameters**		**Number of cases (%)**
**Gender**		
	Female	170 (100%)
	Male	0 (0%)
**Age (years)**		
	< 55	120 (70.59%)
	≥ 55	50 (29.41%)
**T classification**		
	T1	57 (33.53%)
	T2	89 (52.35%)
	T3	18 (10.59%)
	T4	6 (3.53%)
**N classification**		
	LN-	62 (36.47%)
	LN+	108 (63.53%)
**Clinical stage**		
	I	23 (13.53%)
	II	83 (48.82%)
	III	64 (37.65%)
**HER2 targeted therapy**		
	No	78 (45.88%)
	Yes	92 (54.12%)
**Ki67 expression**		
	≤ 30%	77 (45.29%)
	> 30%	93 (54.71%)
**ER Status**		
	Negative	83 (48.82%)
	Positive	87 (51.18%)
**5-year tumor relapse status**		
	No	117 (68.82%)
	Yes	53 (31.18%)
**5-year vital status**		
	Alive	134 (78.82%)
	Dead	36 (21.18%)

Supplementary Table 2. The correlation between DPAGT1 expression and clinicopathological characteristics in HER2 positive breast cancer specimens (n = 170)

	**DPAGT1 expression**		
Characteristics	Low, no. cases (%)	High, no. cases (%)	*P* values
**Age (years)**			
< 55	62 (51.67%)	58 (48.33%)	0.141
≥ 55	32 (64%)	18 (36%)	
**T stage**			
T1	39 (68.42%)	18 (31.58%)	0.014
T2-4	55 (48.67%)	58 (51.32%)	
**N stage**			
N0	42 (67.74%)	20 (32.26%)	0.013
N1	52 (48.15%)	56 (51.85%)	
**Clinical stage**			
I-II	66 (62.26%)	40 (37.74%)	0.019
III	28 (43.75%)	36 (56.25%)	
**ER status**			
Negative	43 (51.81%)	40 (48.19%)	0.372
Positive	51 (58.62%)	36 (41.38%)	
**Ki67 expression**			
≤ 30%	50 (64.94%)	27 (35.06%)	0.021
> 30%	44 (47.31%)	49 (52.69%)	
**5-year tumor relapse status**			
No	81 (69.23%)	36 (30.77%)	< 0.001
Yes	13 (24.53%)	40 (75.47%)	
**5-year vital status**			
Alive	85 (63.43%)	49 (36.57%)	< 0.001
Dead	9 (25%)	27 (75%)	
**ADAM10 expression**			
Low	68 (76.40%)	21 (23.60%)	< 0.001
High	26 (32.10%)	55 (67.90%)	

Supplementary Figure 1

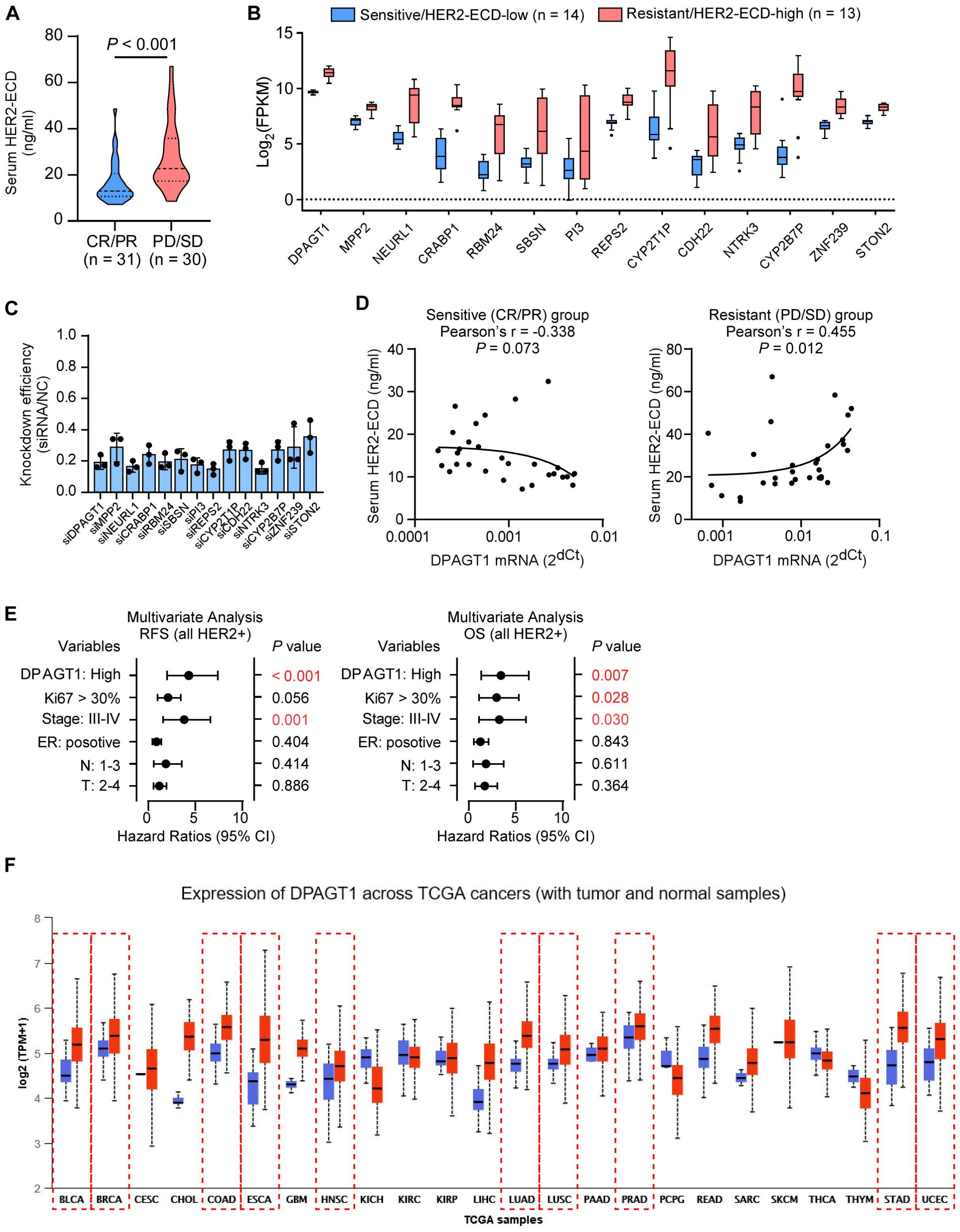

Supplemental Figure 1. (**A**) Quantification of serum HER2-ECD in trastuzumab-sensitive (CR/PR, n = 31) and trastuzumab-resistant (PD/SD, n = 30) HER2+ breast cancer patients. Unpaired two-sided Student's t-test was used. (**B**) RNA-seq analysis showing the expression

of the 14 most significantly upregulated genes in the HER2+ breast cancer tissues with trastuzumab-sensitive/HER2-ECD-low (n = 14) and trastuzumab-resistant/HER2-ECD-high (n = 13). (**C**) Histograms showing the knockdown efficiency of the indicated siRNAs. (**D**) Linear regression analysis of correlation of biopsy DPAGT1 mRNA expression and serum HER2-ECD level in the sensitive (CR/PR) or resistant (PD/SD) groups. (**E**) Multivariate Cox regression analysis of the significant association of high DPAGT1 expression signature and RFS (left) or OS (right) in the presence of other important clinical variables. (**F**) DPAGT1 expression across human cancers in the TCGA data was analyzed by the UALCAN program (http://ualcan.path.uab.edu/analysis.html). Cancers with HER2 overexpression were highlighted with red dashed boxes.

Supplementary Figure 2

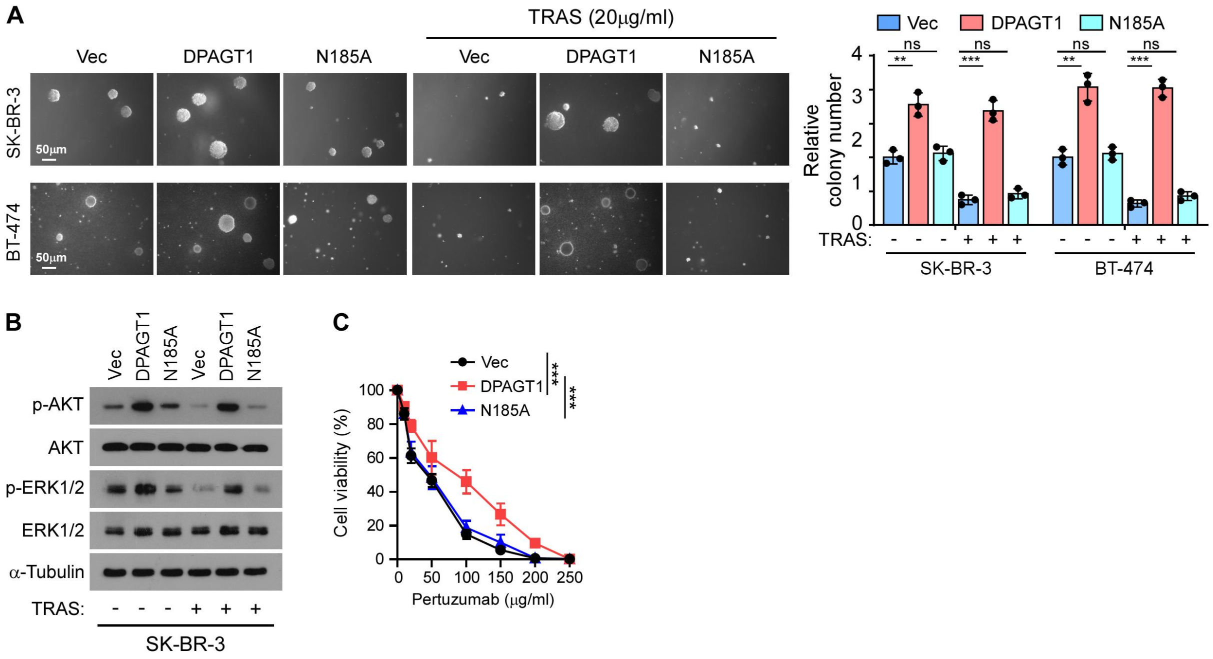

Supplemental Figure 2. (**A**) Representative images (left) and quantification (right) of surviving colonies formed by the vector-, DPAGT1-, or DPAGT1-N185A-transduced SK-BR-3 or BT-474 cells in soft agar upon trastuzumab treatment (20μg/ml). Scale bar: 50 μm. (**B**) IB analysis of expression of HER2, p-AKT, AKT, p-ERK1/2, and ERK1/2 in the indicated SK-BR-3 cells with or without trastuzumab treatment. α-Tubulin was used as a loading control. (**C**) Cell viability assay showing the sensitivity of the vector, DPAGT1, or DPAGT1-N185A expressing SK-BR-3 cells to pertuzumab treatment. Data in (A, C) were plotted as the means ± SD of biological triplicates. Unpaired two-sided Student's t-test was used in (A). Two-way ANOVA was used in (C). *P < 0.05, **P < 0.01, ***P < 0.001, ns, not significant.

Supplementary Figure 3

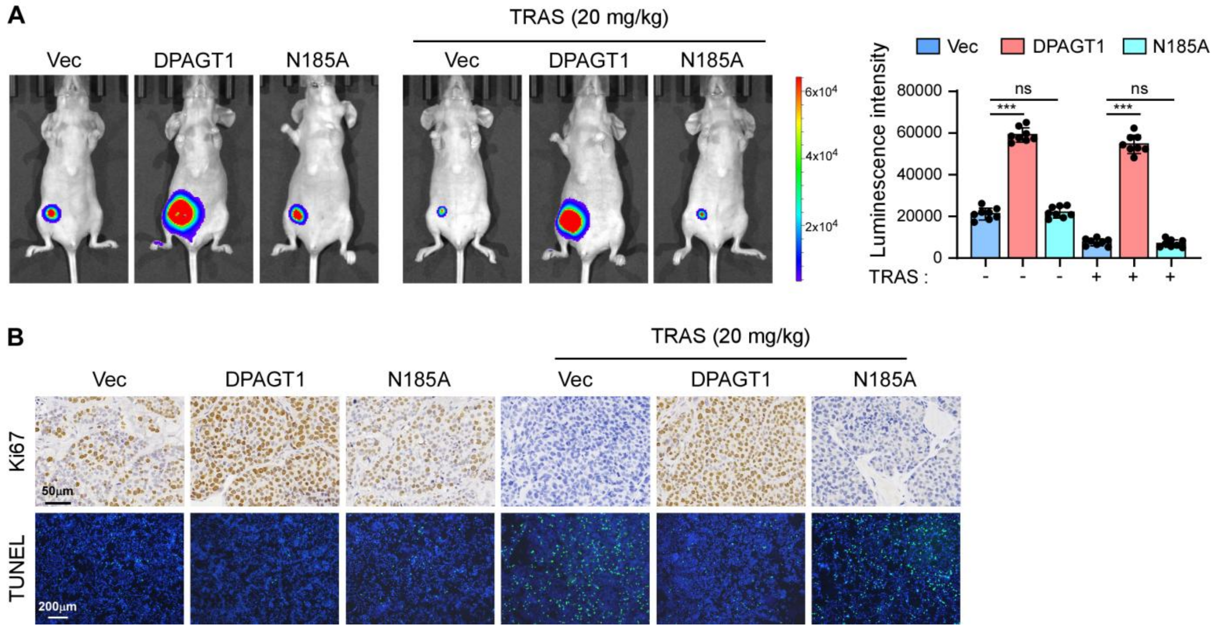

Supplemental Figure 3. (**A**) Representative images (left) and corresponding luciferase signal quantification (right) of vehicle or trastuzumab-treated tumor-bearing mice (n = 8/group). (**B**) Representative image of Ki67 and TUNEL staining in the indicated tumors. Scale bar: 50 µm; 200 µm. Corresponding quantification was shown in Figure 3F. Data in (A) was plotted as the means ± SD of 8 mice. Unpaired two-sided Student's t-test was used in (A). ***P < 0.001, ns, not significant.

Supplementary Figure 4

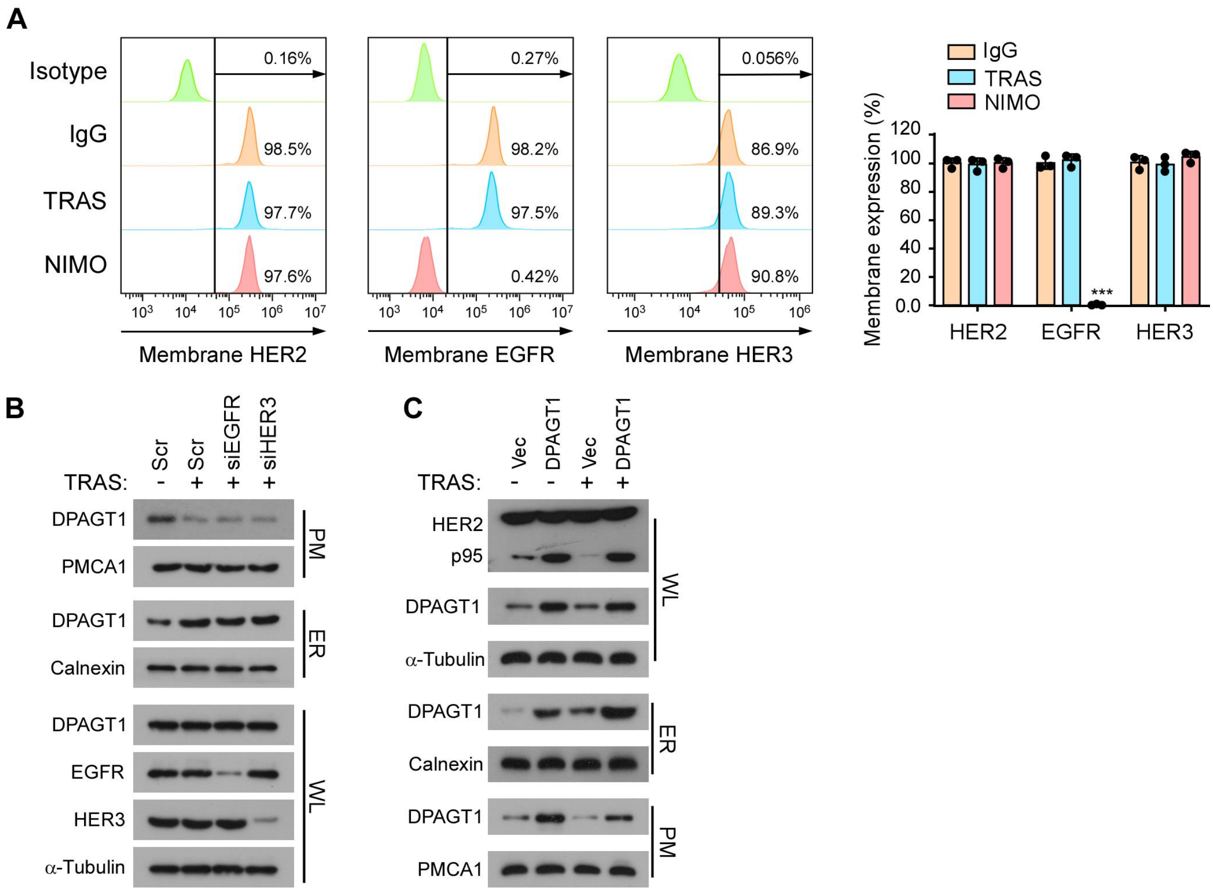

Supplemental Figure 4. (**A**) Flow cytometry analysis (left) and quantification (right) of membrane expression of HER2, EGFR, and HER3 in SK-BR-3 cells with the indicated treatments. Unpaired two-sided Student's t-test was used, ***P < 0.001. (**B**) IB analysis of DPAGT1 expression in the extracted PM, extracted ER, and WL of SK-BR-3 cells transfected with NC, EGFR siRNA, or HER3 siRNA. PMCA1 was used as a loading control of PM. Calnexin was used as a loading control of ER. α-Tubulin was used as a loading control of WL. (**C**) IB analysis of DPAGT1 expression in the extracted PM, extracted ER, and WL of SK-BR-3 and SK-BR-3-DPAGT1 cells with or without TRAS treatment. PMCA1 was used as a loading control of PM. Calnexin was used as a loading control of ER. α-Tubulin was used as a loading control of WL.

Supplementary Figure 5

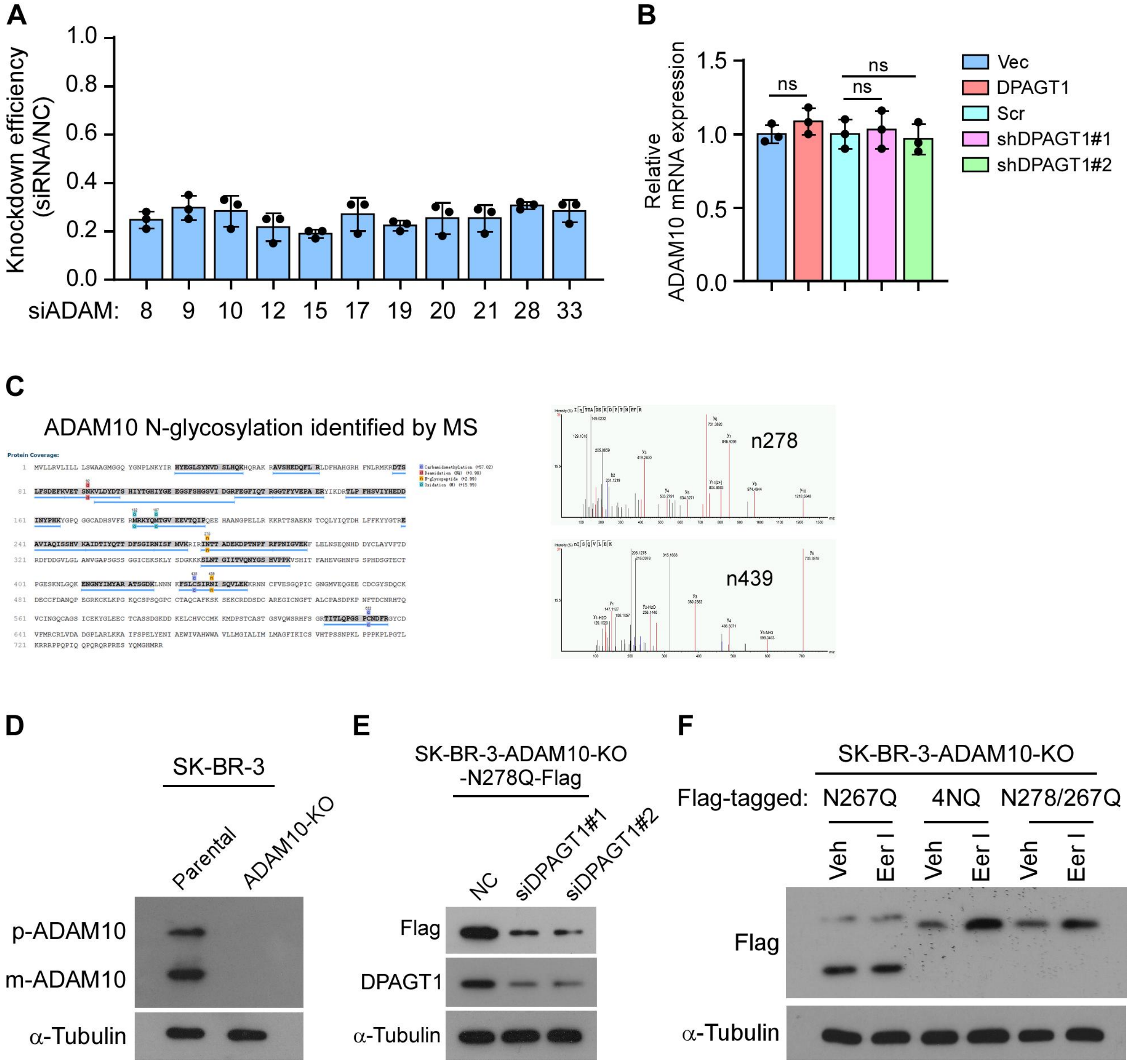

Supplemental Figure 5. (**A**) Histograms showing the knockdown efficiency of the indicated siRNAs. (**B**) Relative expression of ADAM10 mRNA in vector-control, DPAGT1-overexpressing and DPAGT1-silenced SK-BR-3 cells determined by qRT-PCR. (**C**) N-glycosylation modification was identified in the ADAM10 peptides by mass spectrometry analysis of immunoprecipitated ADAM10 in SK-BR-3 cells. (**D**) IB analysis of p- and m-ADAM10 expression in the parental and ADAM10-KO SK-BR-3 cells. α-Tubulin was used as a loading control. (**E**) IB analysis of expression of Flag-tagged ADAM10/N278Q and DPAGT1 in the indicated cells. α-Tubulin was used as a loading control. (**F**) IB analysis of Flag-tagged ADAM10 mutants in the indicated cells treated with or without Eer I. α-Tubulin

was used as a loading control. Data in (A, B) were plotted as the means ± SD of biological triplicates. Unpaired two-sided Student's t-test was used in (B). ns, not significant.

Supplementary Figure 6

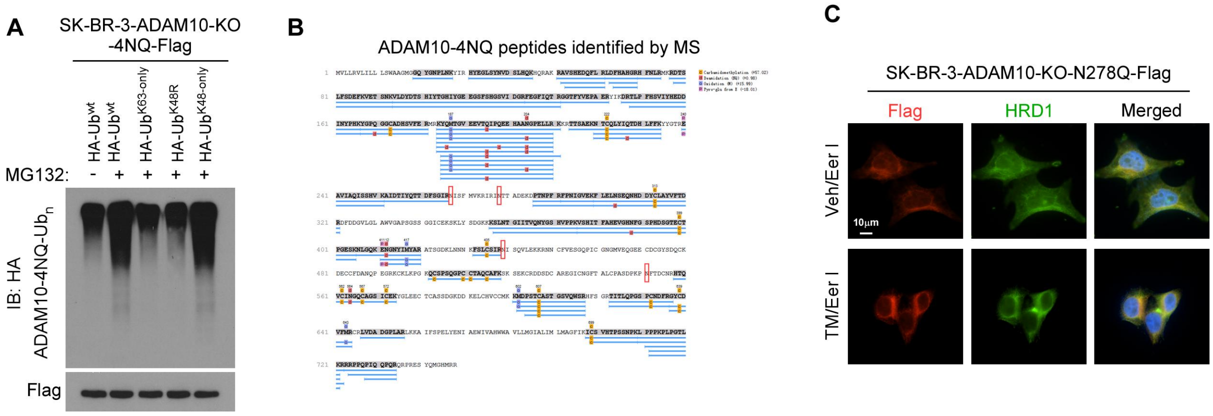

Supplemental Figure 6. (**A**) The 293T cells were transfected with Vector, HA-Ub^{wt}, HA-Ub$^{K63\text{-}only}$, HA-Ub^{K48R}, HA-Ub$^{K48\text{-}only}$, and Flag-ADAM10-4NQ. Cells were treated with MG132 (10 μM) for 6 h before harvest. Lysates were then immunoprecipitated with an anti-Flag antibody, followed by immunoblotting with an anti-HA antibody to examine the ubiquitination level., and with anti-Flag to examine the expression of ADAM10. (**B**) Identified peptides of Flag-ADAM10-4NQ by IP-MS analysis. (**C**) IF staining of Flag and HRD1 in the Flag-ADAM10-N278Q-transduced SK-BR-3-ADAM10-KO cells treated with or without TM and Eer I. Scale bar: 10 μm.

Supplementary Figure 7

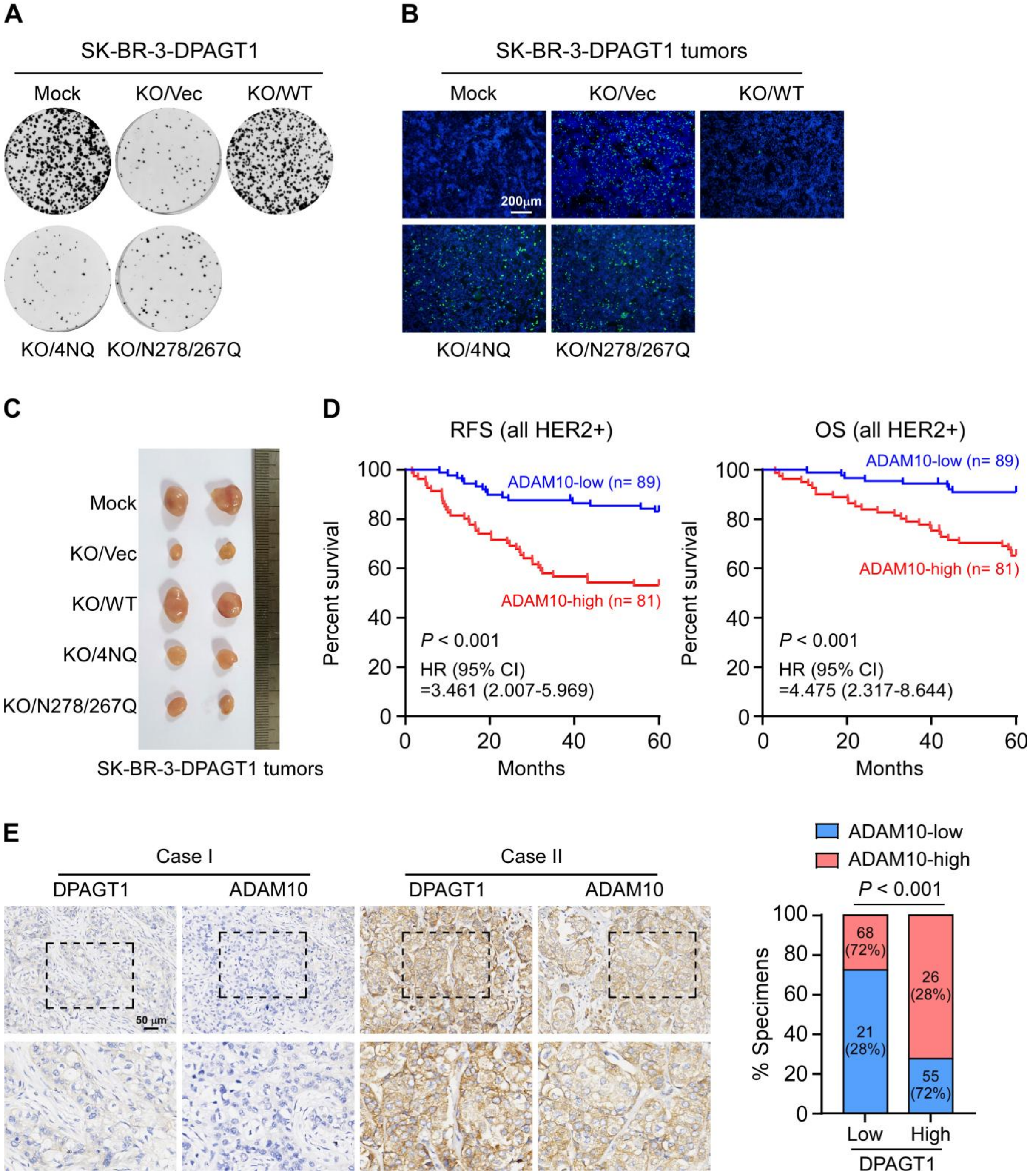

Supplemental Figure 7. (**A**) Representative image of surviving colony formed by the indicated cells. (**B**) Representative image of TUNEL staining in the indicated tumors. Scale bar: 200 μm. Corresponding quantification was shown in Figure 7I. (**C**) Representative images of 2 tumors from each group were shown. (D) Kaplan-Meier analysis of RFS (left) and OS (right) curves in the HER2+ breast cancer patients stratified by ADAM10 expression (n = 170). (E) Representative IHC staining images (left) and positive correlation (right) of

DPAGT1 and ADAM10 in HER2+ breast cancer specimens (n = 170). Scale bar: 50 μm. HR, hazard ratio. Log-rank test was used.

Supplementary Figure 8

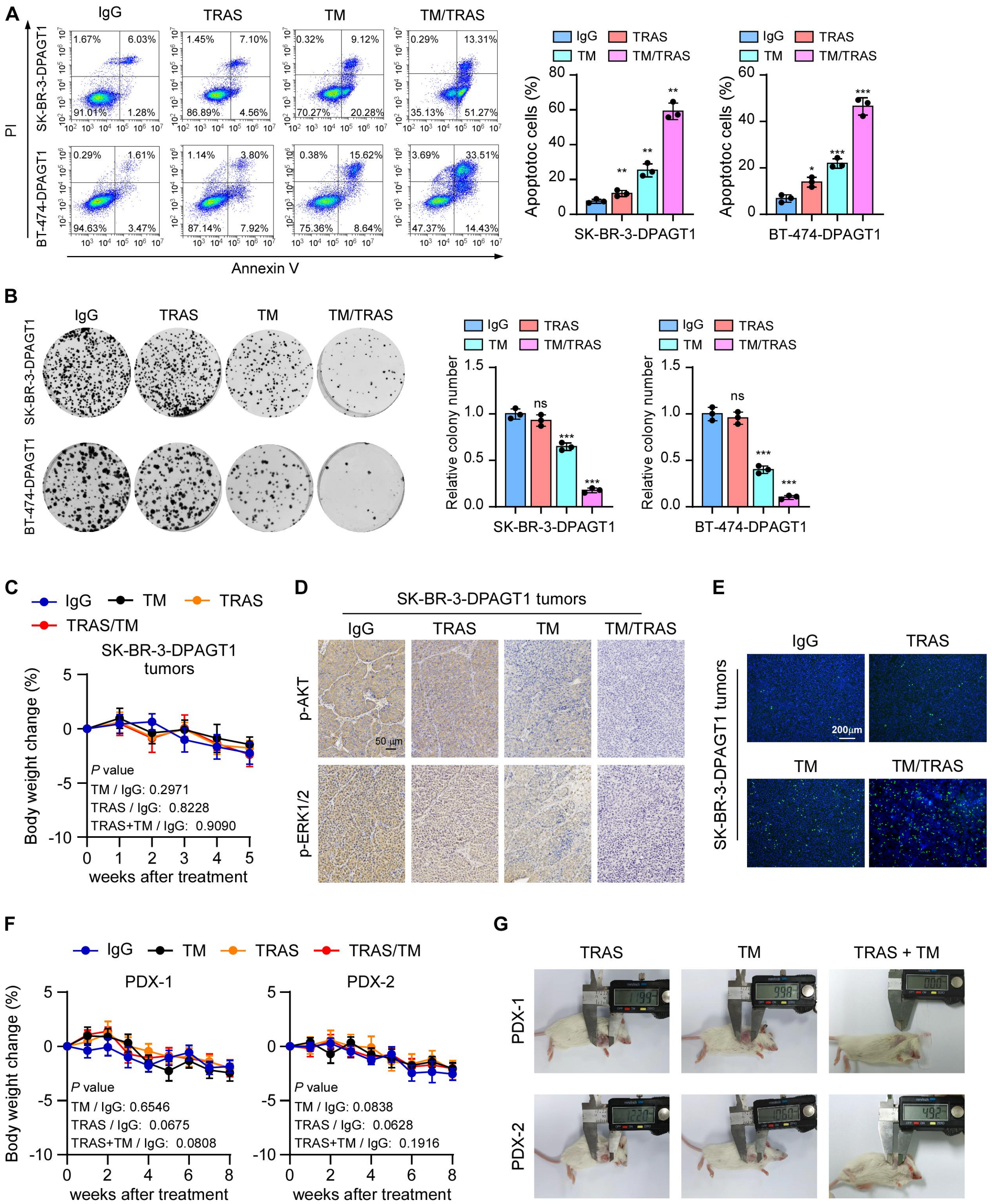

Supplemental Figure 8. (**A**) FACS analysis (left) and quantification (right) of annexin-V staining in SK-BR-3-DPAGT1 cells treated with IgG, or trastuzumab, or TM, or TM plus trastuzumab. (**B**) Representative image (left) and quantification (right) of surviving colony formed by SK-BR-3/DPAGT1 cells with the indicated treatments. (**C**) The body weight curves of the tumor-bearing mice in each indicated treated group. (**D**) IHC staining of p-AKT

and p-ERK1/2 in the indicated SK-BR-3/DPAGT1 tumors. (**E**) Representative image of TUNEL staining in the indicated SK-BR-3/DPAGT1 tumors. Corresponding quantification was shown in Figure 8G. (**F**) The body weight curves of PDX-bearing NOD-SCID mice in each indicated treated group. (**G**) Representative images of PDXs-bearing mice with the indicated treatment. Two-way ANOVA was used in (C, F).
